# Inhibiting methanogenesis by targeting thermodynamics and enzymatic reactions in mixed cultures of rumen microbes *in vitro*

**DOI:** 10.3389/fmicb.2024.1322207

**Published:** 2024-08-14

**Authors:** Kairi Tanaka, Scott Collins, Kathryn Polkoff, Vivek Fellner

**Affiliations:** ^1^Department of Animal Science, North Carolina State University, Raleigh, NC, United States; ^2^Department of Plant and Microbial Biology, North Carolina State University, Raleigh, NC, United States; ^3^Department of Chemical and Biomolecular Engineering, North Carolina State University, Raleigh, NC, United States

**Keywords:** methane mitigation, rumen microbiome, anaerobic fermentation, methanogenesis, thermodynamics, enzymatic reactions

## Abstract

Mitigation of enteric methane (CH_4_) emissions from ruminant livestock represents an opportunity to improve the sustainability, productivity, and profitability of beef and dairy production. Ruminal methanogenesis can be mitigated via two primary strategies: (1) alternative electron acceptors and (2) enzymatic inhibition of methanogenic pathways. The former utilizes the thermodynamic favorability of certain reactions such as nitrate/nitrite reduction to ammonia (NH_3_) while the latter targets specific enzymes using structural analogs of CH_4_ and methanogenic cofactors such as bromochloromethane (BCM). In this study, we investigated the effects of four additives and their combinations on CH_4_ production by rumen microbes in batch culture. Sodium nitrate (NaNO_3_), sodium sulfate (Na_2_SO_4_), and 3-nitro-1-propionate (3NPA) were included as thermodynamic inhibitors, whereas BCM was included as a enzymatic inhibitor. Individual additives were evaluated at three levels of inclusion in experiments 1 and 2. Highest level of each additive was used to determine the combined effect of NaNO_3_ + Na_2_SO_4_ (NS), NS + 3NPA (NSP), and NSP + BCM (NSPB) in experiments 3 and 4. Experimental diets were high, medium, and low forage diets (HF, MF, and LF, respectively) and consisted of alfalfa hay and a concentrate mix formulated to obtain the following forage to concentrate ratios: 70:30, 50:50, and 30:70, respectively. Diets with additives were placed in fermentation culture bottles and incubated in a water bath (39°C) for 6, 12, or 24h. Microbial DNA was extracted for 16S rRNA and ITS gene amplicon sequencing. In experiments 1 and 2, CH_4_ concentrations in control cultures decreased in the order of LF, MF, and HF diets, whereas in experiments 3 and 4, CH_4_ was highest in MF diet followed by HF and LF diets. Culture pH and NH_3_ in the control decreased in the order of HF, MF, to LF as expected. NaNO_3_ decreased (*p* < 0.001) CH_4_ and butyrate and increased acetate and propionate (*p* < 0.03 and 0.003, respectively). Cultures receiving NaNO_3_ had an enrichment of microorganisms capable of nitrate and nitrite reduction. 3NPA also decreased CH_4_ at 6h with no further decrease at 24 h (*p* < 0.001). BCM significantly inhibited methanogenesis regardless of inclusion levels as well as in the presence of the thermodynamic inhibitors (*p* < 0.001) while enriching succinate producers and assimilators as well as propionate producers (*p*_adj_ < 0.05). However, individual inclusion of BCM decreased total short chain fatty acid (SCFA) concentrations (*p* < 0.002). Inhibition of methanogenesis with BCM individually and in combination with the other additives increased gaseous H_2_ concentrations (*p* < 0.001 individually and 0.028 in combination) while decreasing acetate to propionate ratio (*p* < 0.001). Only the cultures treated with BCM in combination with other additives significantly (p_adj_ < 0.05) decreased the abundance of *Methanobrevibacter* expressed as log fold change. Overall, the combination of thermodynamic and enzymatic inhibitors presented a promising effect on ruminal fermentation *in-vitro*, inhibiting methanogenesis while optimizing the other fermentation parameters such as pH, NH_3_, and SCFAs. Here, we provide a proof of concept that the combination of an electron acceptor and a methane analog may be exploited to improve microbial efficiency via methanogenesis inhibition.

## 1 Introduction

Methane (CH_4_) is the key to tackling two of the major obstacles in ruminant nutrition: feed efficiency and greenhouse gas (GHG) emissions. Enteric methane emissions represent up to 10% of dietary energy loss (Blaxter and Clapperton, 1965) and 26.9% of the total anthropogenic CH_4_ emissions in the US (US EPA, 2022). Further, these factors of ruminal methanogenesis may create a negative feedback loop around the price of not only beef and dairy but also other agricultural products because agricultural practices are often dependent on local climate, which is expected to vary drastically due to climate change thereby impacting productivity (Gornall et al., 2010). With the growing public concerns of food insecurity (Capitán-Moyano et al., 2023) and environmental impacts (Myers et al., 2022), mitigation of ruminal methanogenesis is a pivotal point for the future of our agriculture (Beauchemin et al., 2020).

However, methanogenesis serves as a major hydrogen (H_2_) sink to maintain functional microbial fermentation in the rumen (Ungerfeld, 2020). Because of the symbiotic nature of ruminant digestive physiology, merely inhibiting methanogenesis may result in the accumulation of H_2_ thereby inhibiting microbial fermentation and growth (van Soest, 1994). Therefore, ideal means of ruminal methanogenesis inhibition should not only inhibit the methanogenesis pathways but also account for the vacant niche space of H_2_ sink due to the absence of methanogenesis. One strategy to achieve such simultaneous inhibition and replacement would take advantage of the thermodynamics and kinetics of enzymes involved in microbial biochemistry so that thermodynamic inhibitors act as alternative H_2_ sinks while a enzymatic inhibitor directly inactivates a methanogenic molecule.

Thermodynamics dictates the favorability of a reaction while kinetics corresponds to reaction rates, which are governed by the underlying enzymatic mechanisms (Kohn and Boston, 2000; Ungerfeld, 2020). Hence, a thermodynamic inhibitor would be an electron acceptor that competes with methanogenesis for available H_2_ (Kohn and Boston, 2000; Ungerfeld and Kohn, 2006). Conversely, an enzymatic inhibitor exerts its effect by inhibiting an enzyme or cofactor (Ungerfeld and Kohn, 2006). Enzymatic inhibition is reported to be the most efficacious among numerous anti-methanogenic strategies (Chalupa, 1977; Goel et al., 2009; Matsui et al., 2020). Based on the above strategies, we hypothesized that 1) methane inhibition via thermodynamics is dose-dependent whereas methanogenesis inhibition by an enzymatic inhibitor is dose-independent; 2) combinations of thermodynamic inhibitors offer an additive anti-methanogenic effect; and 3) combination of thermodynamic and enzymatic inhibitors inhibit methanogenesis and redistribute H_2_ to other fermentation end products.

Our objective was to determine the effect of four feed additives on CH_4_ production by *in-vitro* cultures of mixed ruminal microbes. Sodium nitrate (NaNO_3_), sodium sulfate (Na_2_SO_4_), and 3-nitro-1-propionate (3NPA) were used as thermodynamic inhibitors, while bromochloromethane (BCM) was used as an enzymatic inhibitor. A total of 4 experiments were conducted. In experiments 1 and 2, individual additives were evaluated at three inclusion levels. In experiment 3 and 4, the highest level of individual additives from previous experiments was used to determine the combined effect of the additives. The effect of additive treatments was evaluated at three different energy levels achieved by varying the forage to concentrate ratio of the basal diets, which consisted of alfalfa pellets and a concentrate mix.

## 2 Materials and methods

### 2.1 Additives and basal diets

Four independent experiments were conducted to test the effectiveness of individual and combined additions of NaNO_3_ (Sigma-Aldrich; purity ≥ 99.0%), Na_2_SO_4_ (EM Science; purity ≥ 99.0), 3NPA (Cayman Chemical Company; purity ≥ 95.0%), and BCM (Chem Service Inc.; purity = 100) on inhibiting CH_4_ in batch cultures of mixed ruminal microbes. Experiments 1 and 2 assessed the effect of independent additives included at four levels (DM basis). The additive treatments were as follows: control (no additive), NaNO_3_ at 7, 14, & 28 g/kg, and Na_2_SO_4_ at 3, 6, & 12 g/kg in experiment 1; and control, 3NPA at 0.5, 1.0, & 2.0 g/kg, and BCM at 0.075, 0.15, & 0.30 g/kg in experiment 2. Doses of NaNO_2_ (Patra and Yu, 2014; Nguyen et al., 2015), Na_2_SO_4_ (van Zijderveld et al., 2010; Patra and Yu, 2014; Gupta et al., 2017), 3NPA (Ochoa-García et al., 2019), and BCM (Tomkins et al., 2009; Abecia et al., 2012) were determined based on literature as cited. Based on the results of experiments 1 and 2, we determined the inclusion levels and combinations for experiments 3 and 4 and assessed the effect of combined additives included at one level as follows: control (no additive); NS(28 g/kg of NaNO_3_, 12 g/kg of Na_2_SO_4_); NSP (NS + 2.0 g/kg of 3NPA); and NSPB (NSP + 0.30 g/kg of BCM). The stock solutions of the above additives were dissolved in deionized H_2_O, except for BCM, which was dissolved in methanol (Supplementary Table S1).

Basal diets consisted of ground alfalfa hay and concentrate mix in three proportions as follows: (1) 70:30 high forage (HF); (2) 50:50 medium forage (MF); and (3) 30:70 low forage (LF). The concentrate mix consisted of a mixture of ground corn, soybean meal, and vitamin and mineral mix. The ingredients and chemical composition of the basal diets are presented in Table 1. Basal diets were designed to provide varying levels of energy from alfalfa and concentrate mix, which averaged 2.28, 2.35, and 2.42 Mcal of ME/kg DM in HF, MF, and LF diets, respectively (National Research Council, 2001). Basal diets (~1.0 g) were quantitatively weighed and placed in 100-mL glass culture bottles in duplicate. The same batches of ground alfalfa and concentrate mix were utilized for all four experiments. On the day of inoculation, respective additive treatments were quantitatively included in the culture bottles. All experiments were repeated twice in separate batch runs for statistical analysis (*n* = 2). For experiments 1 and 2, the first and second batch runs were conducted in summer and fall of 2021, respectively. For experiments 3 and 4, both batch runs were conducted at the same time in winter of 2022.

**Table 1:** Ingredients and chemical composition of three basal diets (high, medium, and low forage) on a dry matter basis (DM).

	**Basal diet** ^*****1*****^		
**Item**	**High forage**	**Medium forage**	**Low forage**
**Feed ingredients, % DM**			
Alfalfa pellets (F)	62.16	44.20	26.40
Concentrate mix (C)^*2*^	26.64	44.20	61.60
Ground corn	21.28	36.14	50.81
Soybean meal	4.48	7.18	9.91
Vitamin and mineral premix	0.89	0.88	0.88
**Chemical composition, % DM** ^*3*^			
Dry matter*, % as-fed*	88.80	88.40	88.00
Metabolizable energy*, Mcal/kg DM*	2.28	2.35	2.42
Net energy*, Mcal/kg DM*	1.72	1.93	2.15
Acid detergent fiber	27.10	20.70	14.20
Neutral detergent fiber	35.90	28.80	21.70
Crude protein^*4*^	18.00	18.10	18.20
Ether extract	3.16	3.46	3.75
Ca	1.20	0.88	0.57
P	0.34	0.36	0.38
Mg	0.28	0.25	0.21
K	2.10	1.80	1.40
Na*, ‰DM*	0.86	0.68	0.51
Cl	0.54	0.42	0.29
S	0.25	0.23	0.20

^*1*^Forage to concentrate ratio for high, medium, and low forage diets are 70:30, 50:50, and 30:70, respectively.

^*2*^Concentrate mix included ground corn, soybean meal, and vitamin and mineral mix, which consisted of 1.2% Zn, 7,800 PPM Mn, 3,380 ppm Cu, 1,300 ppm Fe, 260 ppm I, 78 ppm Se, 26 ppm Co, 750,000 IU/lb vitamin A, 100,000 IU/lb vitamin D3, 5,000 IU/lb vitamin E.

^*3*^Chemical composition of each basal diet was calculated from nutrient values obtained in National Research Council (2001) for alfalfa hay (International Feed #: 1-00-023), ground corn (4-02-854), and soybean meal (5-20-638) unless otherwise stated. The unit for each item is % DM unless specified otherwise.

^*4*^Crude protein contents of ground corn and soybean meal were 16 and 48% DM, respectively.

### 2.2 Rumen fluid collection and inoculation

Rumen contents were collected from a cannulated Hereford steer (*Bos taurus*) fed a basal diet, which consisted predominantly of orchardgrass pasture, throughout the experimental period. The steer was housed at the NCSU Metabolic Unit. The surgery protocol and animal handling procedures were approved by the North Carolina State University Institution of Animal Care and Use Committee (Approval No. 23-163). Whole ruminal contents (approximately 6 L) were obtained 2h postprandially, transported to the lab in pre-heated vacuum containers and squeezed through a double-layered cheesecloth. The strained ruminal fluid was used to inoculate culture bottles. Rumen inoculum was prepared by mixing rumen fluid and artificial saliva in a 1:2 ratio (Gawad and Fellner, 2018). The artificial saliva was prepared according to the ruminant saliva composition as previously outlined by McDougall (1948) and Slyter et al. (1966) and consisted of NaHCO_3_, NaH_2_PO_4_•H_2_O, NaCl, KCl, CaCl_2_•2H_2_O, MgCl_2_•6H_2_O, and Urea. Thirty mL of rumen inoculum were added to each fermentation bottle (nominal volume = 100 mL) that contained 1.0 g of feed substrate and the additive treatments. Culture bottles were flushed with a continuous stream of CO_2_ prior to and during inoculation to maintain anaerobicity. Immediately following the inoculation, the bottles were sealed with rubber-lined septum caps and incubated in a water bath at 39°C. After 0, 6, 12, and 24h of fermentation, respective culture bottles were transferred to an ice bath to terminate further microbial activity. The 12h time point was not measured in experiment 3 and 4 based on experiments 1 and 2.

### 2.3 Chemical measurements

We measured CH_4_, H_2_, pH, ammonia-N (NH_3_), and short-chain fatty acids (SCFAs). At the end of 0, 6, 12, and 24 h, and prior to opening the bottles, a gas sample was taken from the headspace and immediately analyzed for CH_4_ and H_2_. Gas samples (10 μL) were withdrawn directly from the headspace with the aid of a gas tight syringe (Hamilton Co., Reno, NV) and analyzed using a gas chromatograph (model CP-3800; Varian, Walnut Creek, CA) equipped with a stainless-steel column packed with Molsieve 5A 45/60 mesh (Supelco Inc., Bellefonte, PA) as well as the flame ionization detector for the detection of CH_4_ and the thermal conductivity detector for H_2_. Seventy mL of headspace in the bottle allowed for the collection of gas, and the rubber-lined septum cap for retaining the gas in the headspace while taking samples. The expressions of both CH_4_ and H_2_ concentrations (in mM and μM, respectively) are based on the headspace volume of 70 mL (0.07 L). Area counts from 10 μL injections of CH_4_ (770191.76) and H_2_ (98.35) standard gases were used to calculate the concentration (reported as mM for CH_4_ and μM for H_2_) of the respective gas in a sample using the following formula (Equation 1) given the gas constant that 1 mol of gas occupies 22.4 L (and the units were converted from M to mM or μM for CH_4_ and H_2_, respectively):


$$Gas\;\left(M\right)=\frac{area\;count_{sample}}{area\;count_{standard}}\times \frac{mol}{22.4L}$$

After the headspace gas measurement, pH of the culture fluid in the bottle was measured with a pH probe (VWR SympHony—model AR25; Accumet Research, Dual Channel pH/Ion Meter Fisher Scientific). Following pH measurements, culture contents were transferred to a tube and centrifuged at 500 xg for 5 min at 4°C to separate the solid digesta from the liquid. Four mL aliquots of supernatant were transferred to two 5.0-mL centrifuge tubes and kept in a freezer at -80°C for subsequent NH_3_ and SCFA measurements and DNA extraction. After thawing the 4.0-mL aliquots, two 1.0-mL aliquots were transferred into separate microcentrifuge tubes for NH_3_ and SCFA analyses.

Ammonia-N was analyzed using the colorimetric procedure outlined by Beecher and Whitten (1970). Standards containing 0, 4, 8, 12, and 16 μg/mL of NH_3_-N were prepared to generate a standard curve. Culture samples were centrifuged at 21,000 xg for 15 minutes at 4°C to separate any remaining solid particles from the liquid. For the analysis, 5.0 μL of sample or standard were transferred, in duplicate, into glass tubes. Each tube received 100 μL of deionized water, and 0.5 mL of phenol and sodium hypochlorite reagent. The samples and standards were allowed to react for 30 minutes at room temperature. Following 30 min, 4.0 mL of deionized water was added, and the sample was transferred into a cuvette. Absorbance was measured at a wavelength of 630 nm. The standards were used to determine the concentration of unknown samples.

Short chain fatty acids were measured as previously described by Eun et al. (2004) using gas-liquid chromatography (model CP- 3380; Varian, Walnut Creek, CA) equipped with a fused silica capillary column, 30 m × 0.25 mm with 0.25-μm film thickness (NukolTM; Superlco Inc., Bellefonte, PA). One mL aliquots of culture contents were frozen, thawed, and centrifuged at 21,000 xg for 15 min at 4°C to separate remaining solid particles from the liquid. The 1.0-mL sample aliquot was treated with 0.2 mL of a metaphosphoric acid, which included 2-ethylbutyrate as internal standard. The sample was then centrifuged at 21,000 xg for 5 minutes at 4°C, and the supernatant was transferred into a GC vial. The column used in this study detected acetate, propionate, butyrate, valerate, and the isoacids (isobutyrate and isovalerate; we did not measure 2-methylbutyrate, but our results would include it in the isovalerate estimate).

Using the CH_4_, H_2_, and SCFA values measured, theoretical amount of hexose metabolized, production of metabolic hydrogen, recovery of metabolic hydrogen in fermentation end-products and in cells, and the total metabolic hydrogen recovery were calculated using the equation provided by Marty and Demeyer (1973). Additionally, dissolved H_2_ concentrations were estimated using the equation provided by Wang et al. (2016) though the authors highlight the limitation of this estimation. Data were normalized by subtracting the values from the rumen fluid blanks (i.e., rumen inocula without substrates, sacrificed at 0h) from those of samples at 6, 12, and 24h.

### 2.4 DNA sequencing

For DNA sequencing, the following criteria were used to select samples: control and highest level of inclusion; high- and low-forage diets; and incubation of 0 and 24h. This resulted in a total of 88 samples. To facilitate the lysis of microbial cells, an enzymatic lysis mixture was prepared, containing 200 μL of Lytic Enzyme Solution (Qiagen) and 400 μL of MetaPolyzyme (Millipore Sigma MAC4L-5MG), mixed in 19,400 μL of PBS, following the protocol outlined by Maghini et al. (2021). Samples containing 4.0 mL of ruminal fluid were thawed and centrifuged at 12,000 xg for 5 mins at 4°C to separate solid particles from the liquid phase. Supernatant was removed and 200 mL of the enzymatic lysis mixture was added to each sample. The samples were then incubated at 37°C for 1h. After the incubation, microbial DNA was extracted using a Zymo Quick-DNA Fungal/Bacterial Miniprep Kit (Zymo Research), following the manufacturer's protocol.

The concentration of extracted DNA in each sample was measured by UC/Vis spectroscopy using a NanoDrop spectrophotometer (Thermo Fisher Scientific). The DNA concentrations in the samples were standardized to approximately 30 ng/μL of DNA, which was then amplified via polymerase chain reaction (PCR). For PCR, Q5 DNA Polymerase and the following three primer pairs were included separately (i.e. one primer pair per PCR) at 1.25 μL each for forward and reverse primers with Illumina p5 and p7 adapters, respectively (Supplementary Table S2): 515F-806R for universal 16S rRNA gene V4 hypervariable region (Walters et al., 2016); 516F-915R for archaea-specific 16S rRNA gene V4-V5 hypervariable region (Raymann et al., 2017); and ITS3F-ITS4R for fungal ITS2 region (White et al., 1990). For the 515F-806R and 516F-915R primer pairs, the thermocycling conditions included denaturation at 98°C for 15 sec, annealing at 55°C for 20 sec, and extension at 72°C for 15 sec in 35 cycles. For ITS3F-ITS4R primer pair, the thermocycling conditions included denaturation at 98°C for 15 sec, annealing at 65°C for 20 sec, and extension at 72°C for 15 sec in 35 cycles.

The DNA templates were purified with AMPure XP beads (Beckman Coulter, Inc.). Four μL of the purified PCR products were mixed with 25 μL of Q5 DNA Polymerase, 11 μL of nuclease-free H_2_O, and 10 μL of IDT for Illumina DNA/RNA UD Indexes. The prepared libraries were purified with AMPure XP beads (Beckman Coulter, Inc.). The libraries were pooled at 10:1:1 for universal 16S rRNA, archaea-specific 16S rRNA, and fungal ITS2 amplicons, respectively, and sequenced on Illumina MiSeq using a MiSeq v3 2x300bp paired end flow cell (20M reads) in the Genomic Sciences Laboratory at North Carolina State University.

### 2.5 Bioinformatics

Sequence data were first processed using Cutadapt (Martin, 2011) to separate reads into three separate FASTQ files based on the primer sequence present, resulting in three sets of sequence reads: universal 16S, archaea-specific 16S, and fungal ITS datasets. A custom bash script was created to run the Cutadapt program and to count the number of reads in each of the original and new FASTQ files. Each dataset was then processed following the DADA2 pipeline (Callahan et al., 2016) in R version 4.3.0 (R Core Team, 2023) using RStudio (Posit team, 2023) to infer amplicon sequence variants (ASVs) and assign taxonomy, i.e. the standard DADA2 pipeline for universal and archaea-specific 16S amplicon sequences and the ITS DADA2 pipeline for fungal ITS2 amplicon sequences. For ASVs in the universal and archaea-specific 16S datasets, Silva version 138 reference database was utilized (Quast et al., 2013; Yilmaz et al., 2014). For ASVs in the fungal ITS dataset, UNITE general FASTA release for Fungi 2 was used (Abarenkov et al., 2022).

The ASV and taxa tables from the three separate runs through the DADA2 pipelines were merged for sequence alignment and phylogenetic information inference using the *DECIPHER* (Wright, 2016) and *phangorn* (Schliep, 2011) packages, respectively. The NCSU High Performance Computing Hazel Cluster was utilized for this step due to the computational requirements. The ASV, taxonomy assignment, sequence, and phylogenetic information was compiled into a *phyloseq* (McMurdie and Holmes, 2013) object for downstream analyses.

For alpha diversity, Chao1 diversity, Shannon diversity, and Simpson diversity indices were estimated on the ASV level. For further downstream analyses, ASVs were filtered to the abundance and prevalence of more than 3 (i.e. ASVs only present in more than 3 counts and samples). Beta diversity was estimated on centered-log ratio transformed abundance data using the Bray-Curtis dissimilarity and weighted UniFrac distance as measurements of dissimilarity. To ordinate these dissimilarity and distance measurements, principal coordinate analysis (PCoA) was performed.

From the filtered sequences, pathway abundances were predicted using PICRUSt2 (Douglas et al., 2020). The predicted metagenomic functional data were imported into *R* and used as a *TreeSummarizedExperiment* (Huang et al., 2021) object for statistical analysis.

### 2.6 Statistical analysis

Data from above measurements and estimations were analyzed according to a completely randomized block design using a mixed model using the following R packages: *lme4* (Bates et al., 2015), *lmerTest* (Kuznetsova et al., 2017), and *emmeans* (Lenth, 2023) including polynomial contrasts for linear and quadratic trend of the level effect. Data from 6, 12, and 24h were analyzed separately. Thus, the model included treatment and diet as fixed effects while the replicate variable nested within the batch run as a random effect. Thus, the model is represented by:


(1) $$\begin{array}{l} y_{ijkl}=\beta_{0}+\beta_{i}x_{i}+\beta_{j}x_{j}+\beta_{ij}x_{ij}+\gamma_{k\left(l\right)}+\epsilon_{ijkl} \end{array}$$

where

*y*_*ijkl*_ = each response variable measured,

β_0_ = overall mean,

β_*i*_*x*_*i*_ = fixed effect of additive level or treatment,

β_*j*_*x*_*j*_ = fixed effect of diet,

β_*ij*_*x*_*ij*_ = interaction term of additive level or treatment and diet fixed effects,

γ_*k*(*l*)_ = random effect of replicate nested within run,

ϵ_*ijkl*_ = residuals.

For alpha and beta diversity, the *RRPP* package (Collyer and Adams, 2018) was used for permutational analysis of variance (PERMANOVA). To analyze the relative abundance of archaeal taxa, untransformed abundance data were transformed to relative terms, which were then used as the response variable in the above mixed model. For differential abundance analysis of microbial taxa as well as predicted pathways, the *ANCOMBC* package was used to conduct the analysis of compositions of microbiomes with bias correction 2 (ANCOM-BC2) (Lin and Peddada, 2020; Lin et al., 2022). Only the individual fixed effects of treatment and diet were included in the model for this analysis because the interaction term could not be included due to the sample size requirement of the ANCOM-BC2 algorithm. The Benjamini-Hochberg adjustment was used as a p-value adjustment method (p_adj_) for multiple hypothesis tests.

Significant effects were declared at p-value ≤ 0.05 and tendencies at p-value ≤ 0.10. The analyzed data were visualized in figures using the following R packages: *tidyverse* (Wickham et al., 2019) and *ggpubr* (Kassambara, 2023).

## 3 Results

### 3.1 Experiment 1—*In-vitro* fermentation profile

**Figure 1:**
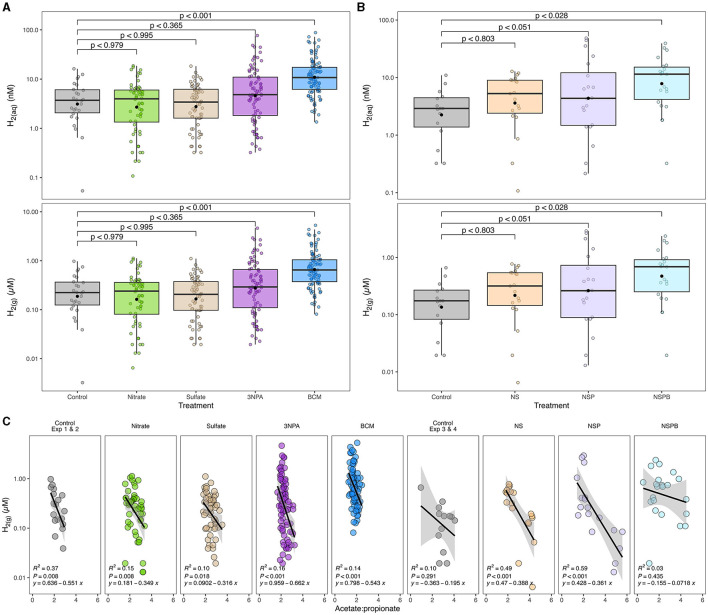
Effects of individual **(A)** and combined **(B)** sodium nitrate (Nitrate; N), sodium sulfate (Sulfate; S), 3-nitro-1-propionate (3NPA; P), and bromochloromethane (BCM; B) on gaseous and estimated dissolved hydrogen concentration (H_2(g)_ μM and H_2(aq)_ nM, respectively) and its correlation with acetate-to-propionate ratio **(C)**. Dissolved H_2_ was estimated as outlined in Wang et al. (2016). In **(A, B)**, the black filled point within each box plot represents the mean. The y-axes of the three figures are presented on log10 scale. Data are representative of all incubation periods (6, 12, and 24h) and diet (varying forege:concentrate, 70:30, 50:50, and 30:70) with rumen microbes in an *in-vitro* mixed batch culture system.

Effects of diets and additives in experiment 1 are reported in Tables 2, 3, Supplementary Tables S3–S6, Figure 1A. There was an interaction between time and treatment (*p* < 0.001) in all experiments; results are shown by time. The pH values in control cultures ranged from 5.9 at 6 h to 5.2 at 24 h. Total SCFA concentrations at 24h were similar (*p* > 0.10) in control cultures (Table 3). Acetate decreased and butyrate increased as dietary concentrate increased (Table 3). But proportions of propionate were not affected (*p* > 0.10). Molar proportions of valerate and isoacids were not affected by diet.

**Table 2:** Effect of sodium nitrate (NaNO_3_) on methane (CH_4_), pH and ammonia (NH_3_) after 6, 12, and 24h of incubation with rumen microbes in an *in-vitro* mixed batch culture system fed varying forege:concentrate (HF = 70:30, MF=50:50, and LF = 30:70) in experiment 1.

	**Diet** ^*****1*****^														
	**HF**				**MF**				**LF**						
	**NaNO**_**3**_, ***g/kg DM***													***p*** **<**		
**Item**	**0**	**7**	**14**	**28**	**0**	**7**	**14**	**28**	**0**	**7**	**14**	**28**	**SE**	**D^*2*^**	**L^*3*^**	**DxL^*4*^**
**6 Hr**																
CH_4_*, mM*	1.33	0.54	0.27	0.02	1.19	0.64	0.30	0.13	1.48	1.02	0.32	0.18	0.15	0.020	0.001	0.350
pH	5.87	6.38	6.02	6.73	5.82	6.19	6.21	6.09	5.87	5.93	6.15	6.15	0.09	0.003	0.001	0.001
NH_3_*, mM*	5.25	6.87	8.37	5.33	5.63	6.71	5.59	6.71	3.92	4.52	6.38	6.94	1.44	0.400	0.120	0.190
**12 Hr**																
CH_4_*, mM*	1.98	1.53	0.89	0.14	2.28	1.42	1.10	0.14	1.91	1.67	1.21	0.13	0.52	0.790	0.001	0.860
pH	5.54	5.67	5.72	5.92	5.52	5.53	5.68	5.94	5.50	5.52	5.50	5.90	0.06	0.004	0.001	0.140
NH_3_*, mM*	4.92	6.47	6.35	7.26	6.57	6.52	7.80	6.68	4.57	5.17	5.30	6.97	1.18	0.110	0.190	0.750
**24 Hr**																
CH_4_*, mM*	1.66	1.71	1.01	0.05	1.81	1.86	1.25	0.11	2.49	2.16	1.53	0.22	0.53	0.004	0.001	0.730
pH	5.21	5.30	5.35	5.57	5.21	5.25	5.28	5.52	5.12	5.18	5.23	5.40	0.04	0.001	0.001	0.290
NH_3_*, mM*	5.31	5.56	5.63	8.17	5.03	5.15	5.26	7.50	4.96	4.98	5.40	6.66	2.50	0.240	0.001	0.940

^*1*^HF, high forage; MF, medium forage; LF, low forage.

^*2*^Diet effect.

^*3*^Treatment level effect.

^*4*^Diet and level interaction.

**Table 3:** Effect of sodium nitrate (NaNO_3_) on short chain fatty acids (SCFA) after 6, 12, and 24h of incubation with rumen microbes in an *in-vitro* mixed batch culture system fed varying forege:concentrate (HF = 70:30, MF = 50:50, and LF = 30:70) in experiment 1.

	**Diet** ^*****1*****^														
	**HF**				**MF**				**LF**						
	**NaNO**_**3**_, ***g/kg DM***													***p*** **<**		
**Item**	**0**	**7**	**14**	**28**	**0**	**7**	**14**	**28**	**0**	**7**	**14**	**28**	**SE**	**D^*2*^**	**L^*3*^**	**DxL^*4*^**
**6 Hr**																
Total SCFA*, mM*	83.87	88.24	81.71	52.26	84.65	82.95	74.74	61.24	72.08	71.85	61.36	52.66	12.70	0.220	0.020	0.95
Acetate (A)*, mol%*	66.84	68.72	69.29	68.15	64.54	67.00	68.40	70.85	62.09	62.97	68.80	72.33	2.07	0.450	0.003	0.20
Propionate (P)*, mol%*	24.99	23.06	23.87	27.59	25.72	24.55	23.61	22.51	26.59	26.76	22.09	19.69	2.40	0.730	0.370	0.22
Butyrate*, mol%*	6.99	6.92	5.71	2.71	8.26	6.60	6.14	5.00	9.04	8.19	7.17	5.86	0.96	0.010	0.001	0.64
Valerate*, mol%*	0.96	0.86	0.75	1.01	1.09	0.96	0.98	0.81	1.31	1.18	1.05	1.14	0.17	0.060	0.430	0.86
Isoacids*, mol%*	0.22	0.43	0.37	0.38	0.39	0.43	0.40	0.35	0.50	0.42	0.42	0.49	0.37	0.840	0.990	1.00
A:P	2.68	3.02	2.91	2.68	2.54	2.72	2.89	3.14	2.32	2.36	3.10	3.66	0.29	0.970	0.030	0.11
**12 Hr**																
Total SCFA*, mM*	110.67	112.29	112.01	95.70	100.14	108.54	111.39	89.27	103.60	99.74	112.28	94.53	9.10	0.080	0.001	0.36
Acetate (A)*, mol%*	62.85	64.09	65.32	69.65	61.45	61.80	63.08	68.49	59.04	59.81	58.41	67.44	2.00	0.001	0.001	0.12
Propionate (P)*, mol%*	27.01	25.33	25.47	23.95	26.74	27.55	25.77	24.08	26.47	25.94	26.89	24.64	3.25	0.590	0.010	0.67
Butyrate*, mol%*	8.69	9.06	7.73	5.29	10.35	9.53	9.81	6.20	12.88	12.82	13.20	6.74	1.42	0.001	0.001	0.03
Valerate*, mol%*	1.02	1.12	1.04	0.71	1.18	0.94	1.07	0.81	1.21	1.12	1.15	0.77	0.12	0.340	0.001	0.54
Isoacids*, mol%*	0.42	0.40	0.44	0.40	0.29	0.18	0.27	0.41	0.39	0.31	0.35	0.41	0.14	0.001	0.040	0.29
A:P	2.42	2.59	2.62	2.93	2.35	2.39	2.49	2.86	2.27	2.34	2.21	2.77	0.38	0.003	0.001	0.75
**24 Hr**																
Total SCFA*, mM*	134.72	147.29	148.06	134.93	138.63	140.94	139.10	128.74	129.56	132.66	140.51	131.89	3.51	0.020	0.002	0.27
Acetate (A)*, mol%*	58.33	59.92	59.24	62.20	55.27	55.95	56.08	58.68	48.54	49.18	49.41	51.49	1.35	0.001	0.030	1.00
Propionate (P)*, mol%*	26.84	25.71	27.32	28.04	27.69	26.37	26.95	28.49	26.93	25.57	26.63	29.11	2.10	0.760	0.003	0.92
Butyrate*, mol%*	13.34	12.79	11.75	8.39	15.61	16.24	15.48	11.28	22.70	23.21	22.03	17.41	2.47	0.001	0.001	1.00
Valerate*, mol%*	1.10	1.18	1.13	1.00	1.02	1.03	1.11	1.23	1.28	1.42	1.30	1.39	0.16	0.001	0.520	0.17
Isoacids*, mol%*	0.39	0.39	0.56	0.37	0.41	0.41	0.38	0.32	0.55	0.62	0.63	0.59	0.12	0.001	0.420	0.72
A:P	2.18	2.34	2.18	2.23	2.00	2.14	2.10	2.07	1.82	2.01	1.87	1.77	0.17	0.001	0.150	0.96

^*1*^HF, high forage; MF, medium forage; LF, low forage.

^*2*^Diet effect.

^*3*^Treatment level effect.

^*4*^Diet and level interaction.

At 6h, there was an interaction (*p* < 0.001) between inclusion level of NaNO_3_ and diet on culture pH. At 24h, NaNO_3_ decreased (*p* < 0.01) CH_4_ irrespective of diet. The decrease was more than 90% and greatest at the highest level of inclusion. In contrast, NaNO_3_ increased (*p* < 0.01) culture pH and NH_3_ irrespective of diet (Table 2).

Sodium nitrate inclusion levels increased acetate and valerate and decreased butyrate in a linear manner (all at *p* < 0.001). Nitrate supplementation also had a linear effect (*p* < 0.004) on total SCFA that increased at the 7 and 14 g/kg DM inclusion levels and remained similar to the control at the 28 g/kg DM level. Acetate molar proportions increased linearly (*p* < 0.001) while butyrate molar proportions decreased linearly (*p* < 0.001). Molar proportions of propionate increased at the 28 g/kg DM inclusion level of NaNO_3_ (*p* < 0.005; Table 3).

Sodium sulfate had a diet by treatment interaction with respect to CH_4_, pH, and NH_3_ at 24h (*p* < 0.02, 0.05, and 0.04, respectively). Methane decreased in HF diet and increased in MF but in LF diets, the 3 g/kg DM level increased CH_4_ while the 6 and 12 g/kg DM levels decreased CH_4_ (Supplementary Table S3). Culture pH and NH_3_ increased in HF diet and decreased in MF and LF diets. Propionate decreased (*p* < 0.04) with the inclusion of 6 and 12 g/kg DM of Na_2_SO_4_ inclusion, which was also reflected in increased A:P (*p* < 0.02; Supplementary Table S4). Other SCFAs were not affected (*p* > 0.10).

The levels of NaNO_3_ inclusion had a linear effect (*p* < 0.04) on theoretical amounts of hexose metabolized at 6 and 12h (*p* < 0.04), on metabolic hydrogen recovered in end-products at 24h (*p* < 0.001), on cellular metabolic hydrogen recovery at 12 and 24h (*p* < 0.001) and total metabolic hydrogen recovery at 24h (*p* < 0.001; Supplementary Table S5).

The inclusion levels of Na_2_SO_4_ had a linear effect on theoretical hexose metabolism at 24h (*p* < 0.004), total metabolic hydrogen production at 12 and 24h (*p* < 0.01 and 0.001), end-product hydrogen recovery at 24h (*p* < 0.001), cellular metabolic hydrogen recovery at 6 and 24 h (*p* < 0.001), and total metabolic hydrogen recoveries at 24h (*p* < 0.001). Quadratic effects of sodium sulfate inclusion levels were observed for total metabolic hydrogen production at 6h (*p* < 0.03) and cellular metabolic hydrogen recovery at 12h (*p* < 0.02; Supplementary Table S6).

### 3.2 Experiment 2—*In-vitro* fermentation profile

Effects of diets and additives in experiment 2 are reported in Tables 4, 5, Supplementary Tables S7–S10, and Figure 1A. There was an interaction between 3NPA and diet at 24h for CH_4_ and pH (*p* < 0.005 and 0.03, respectively). In the HF diet, 0.5 g/kg of 3NPA decreased CH_4_ by 63% and increased pH compared to the MF and LF diets and the control. In contrast, 0.5 g/kg of 3NPA increased CH_4_ at the 0.5 g/kg DM level with MF diet but decreased CH_4_ at the 2.0 g/kg level with MF and LF diet (Supplementary Table S7). At 3NPA inclusion of 0.5 g/kg in HF, at 2.0 g/kg in MF and LF diets, culture pH was the highest within the diet. Total SCFA concentration decreased linearly (*p* < 0.002) with the 3NPA inclusion levels (Supplementary Table S8). Propionate decreased linearly (*p* < 0.02), which in turn linearly increased A:P (*p* < 0.001). Valerate increased (*p* < 0.01), and other SCFAs were not affected. At 6h, molar proportions of butyrate and valerate decreased linearly and quadratically (*p* < 0.001 and 0.004, respectively).

**Table 4:** Effect of bromochloromethane (BCM) on methane (CH_4_), pH and ammonia (NH_3_) after 6, 12, and 24h of incubation with rumen microbes in an *in-vitro* mixed batch culture system fed varying forege:concentrate (HF = 70:30, MF = 50:50, and LF = 30:70) in experiment 2.

	**Diet** ^*****1*****^														
	**HF**				**MF**				**LF**						
	**BCM**, ***g/kg DM***													***p*** **<**		
**Item**	**0**	**0.075**	**0.15**	**0.3**	**0**	**0.075**	**0.15**	**0.3**	**0**	**0.075**	**0.15**	**0.3**	**SE**	**D^*2*^**	**L^*3*^**	**DxL^*4*^**
**6 Hr**																
CH_4_*, mM*	1.33	0.05	0.02	0.02	1.19	0.05	0.02	0.02	1.48	0.05	0.03	0.02	0.07	0.320	0.001	0.380
pH	5.87	5.80	5.87	5.85	5.82	5.83	5.87	5.87	5.87	5.85	5.85	5.90	0.02	0.340	0.050	0.300
NH_3_*, mM*	5.25	7.20	6.53	6.76	5.63	6.24	6.79	6.95	5.92	7.28	6.65	7.96	1.20	0.410	0.030	0.820
**12 Hr**																
CH_4_*, mM*	1.98	0.02	0.01	0.01	2.28	0.03	0.01	0.01	1.91	0.01	0.01	0.01	0.25	0.770	0.001	0.960
pH	5.54	5.51	5.46	5.48	5.52	5.46	5.43	5.39	5.49	5.31	5.27	5.37	0.03	0.001	0.001	0.010
NH_3_*, mM*	4.92	6.11	5.73	6.18	6.57	5.80	5.48	6.12	4.57	4.08	4.39	4.41	1.26	0.010	0.940	0.810
**24 Hr**																
CH_4_*, mM*	1.66	0.02	0.01	0.00	1.81	0.01	0.00	0.00	2.49	0.02	0.00	0.00	0.22	0.220	0.001	0.170
pH	5.21	5.32	5.34	5.33	5.21	5.23	5.24	5.21	5.12	5.18	5.20	5.16	0.01	0.001	0.001	0.001
NH_3_*, mM*	5.31	5.51	7.53	4.91	5.03	5.93	5.22	5.70	4.96	4.66	2.18	3.41	1.45	0.010	0.840	0.220

^*1*^HF, high forage; MF, medium forage; LF, low forage.

^*2*^Diet effect.

^*3*^Treatment level effect.

^*4*^Diet and level interaction.

**Table 5:** Effect of bromochloromethane (BCM) on short chain fatty acids (SCFA) after 6, 12, and 24h of incubation with rumen microbes in an *in-vitro* mixed batch culture system fed varying forege:concentrate (HF = 70:30, MF = 50:50, and LF = 30:70) in experiment 2.

	**Diet** ^*****1*****^														
	**HF**				**MF**				**LF**						
	**BCM**, ***g/kg DM***													***p*** **<**		
**Item**	**0**	**0.075**	**0.15**	**0.3**	**0**	**0.075**	**0.15**	**0.3**	**0**	**0.075**	**0.15**	**0.3**	**SE**	**D^*2*^**	**L^*3*^**	**DxL^*4*^**
**6 Hr**																
Total SCFA*, mM*	83.87	81.83	82.10	81.95	84.65	75.63	64.89	60.09	79.13	70.71	76.60	68.42	7.24	0.060	0.230	0.61
Acetate (A)*, mol%*	66.84	62.08	62.04	62.18	64.54	58.21	60.00	60.67	61.80	59.23	61.25	62.67	0.99	0.001	0.001	0.14
Propionate (P)*, mol%*	24.99	30.03	29.59	29.34	25.72	31.16	30.73	30.61	24.98	29.23	28.63	28.39	1.79	0.010	0.001	0.98
Butyrate*, mol%*	6.99	6.81	7.24	7.31	8.26	9.18	8.11	7.73	10.89	10.49	7.94	7.79	1.55	0.002	0.120	0.22
Valerate*, mol%*	0.96	0.86	0.93	0.98	1.09	1.23	1.22	1.17	1.46	1.11	1.35	1.03	0.20	0.001	0.260	0.09
Isoacids*, mol%*	0.22	0.23	0.20	0.19	0.39	0.22	-0.07	-0.18	0.76	-0.05	0.88	0.11	0.23	0.120	0.120	0.13
A:P	2.68	2.07	2.10	2.12	2.54	1.89	1.96	1.99	2.44	2.05	2.15	2.22	0.14	0.020	0.001	0.48
**12 Hr**																
Total SCFA*, mM*	110.67	108.30	109.66	121.18	100.14	93.13	98.87	100.04	103.61	96.23	89.84	92.17	4.77	0.001	0.100	0.07
Acetate (A)*, mol%*	62.85	58.77	57.63	56.76	61.45	55.53	54.96	54.77	59.04	52.41	52.31	52.14	0.89	0.001	0.001	0.87
Propionate (P)*, mol%*	27.01	30.05	30.68	31.35	26.74	32.36	31.64	31.14	26.47	30.02	29.98	30.12	1.84	0.130	0.001	0.79
Butyrate*, mol%*	8.69	9.86	10.34	10.50	10.35	10.86	12.12	12.82	12.88	16.22	16.52	16.37	1.54	0.001	0.001	0.29
Valerate*, mol%*	1.02	1.21	1.25	1.23	1.18	1.26	1.24	1.27	1.21	1.28	1.26	1.31	0.18	0.440	0.330	0.98
Isoacids*, mol%*	0.42	0.11	0.10	0.16	0.29	0.00	0.04	0.00	0.39	0.07	-0.06	0.06	0.08	0.110	0.001	0.89
A:P	2.42	1.96	1.88	1.82	2.35	1.72	1.74	1.76	2.27	1.75	1.75	1.73	0.16	0.120	0.001	0.98
**24 Hr**																
Total SCFA*, mM*	134.72	108.69	113.60	128.82	138.63	116.96	117.32	117.55	129.56	110.03	122.17	119.41	7.40	0.890	0.002	0.67
Acetate (A)*, mol%*	58.33	51.42	50.99	50.56	55.27	48.07	47.02	46.33	48.54	46.66	45.27	44.60	3.03	0.001	0.001	0.50
Propionate (P)*, mol%*	26.84	29.60	30.61	30.68	27.69	30.48	30.58	31.27	26.93	31.11	29.80	31.03	1.31	0.160	0.001	0.27
Butyrate*, mol%*	13.34	17.14	16.72	16.79	15.61	19.73	20.66	20.52	22.70	20.72	22.90	22.41	3.69	0.001	0.030	0.25
Valerate*, mol%*	1.10	1.69	1.61	1.65	1.02	1.52	1.58	1.65	1.28	1.41	1.83	1.72	0.46	0.510	0.001	0.63
Isoacids*, mol%*	0.39	0.15	0.07	0.33	0.41	0.20	0.16	0.22	0.55	0.09	0.20	0.24	0.10	0.890	0.001	0.75
A:P	2.18	1.74	1.67	1.65	2.00	1.57	1.53	1.48	1.82	1.49	1.52	1.43	0.06	0.001	0.001	0.76

^*1*^HF, high forage; MF, medium forage; LF, low forage.

^*2*^Diet effect.

^*3*^Treatment level effect.

^*4*^Diet and level interaction.

Methane linearly decreased with the addition of BCM at 6, 12, and 24h (at least **p** < 0.01; Table 4). At 12 and 24h, there was an interaction (*p* < 0.01 and 0.001, respectively) between inclusion level of BCM and diet on pH. Culture pH increased quadratically at 24h (*p* < 0.04) and linearly at 12 h (*p* < 0.001). Concentration of NH_3_ increased linearly with the BCM inclusion levels at 6 and 24h (*p* < 0.05 and 0.005, respectively) and quadratically at 12h (*p* < 0.05). Concomitantly, H_2_ in the BCM treatment increased (*p* < 0.001) when averaged across inclusion level compared to control (Figure 1A).

Total SCFA decreased (*p* < 0.002) with the addition of BCM at 24 h (Table 5). Molar proportion of acetate decreased (*p* < 0.001) while that of butyrate (*p* < 0.001) and propionate (*p* < 0.04) increased with an increase in the level of BCM inclusion. Consequently, the A:P ratio was decreased (*p* < 0.04) with an increase in the level of BCM inclusion. BCM increased (*p* < 0.05) valerate and decreased (*p* < 0.001) isoacids.

The levels of 3NPA had a linear effect on hexose metabolism at 6h (*p* < 0.04), total metabolic hydrogen production at 12 and 24 h (*p* < 0.001), end-product hydrogen recovery at 12 and 24 h (*p* < 0.01 and 0.001), cellular hydrogen recovery across all time points (*p* < 0.01, 0.001, and 0.001), and total hydrogen recovery at 24 h. The 3NPA levels exhibited quadratic effects on total metabolic hydrogen production and recovery (*p* < 0.01) at 6 h (Supplementary Table S9).

BCM had linear effects on hexose metabolism at 6 and 12 h (*p* < 0.05 and 0.002), end-product metabolic hydrogen recovery at 12 and 24 h (*p* < 0.001) and cellular metabolic hydrogen recovery at 24 h (*p* < 0.001) as well as quadratic effects on total metabolic hydrogen production at 6 and 12 h (*p* < 0.01 and 0.02), end-product metabolic hydrogen recovery at 6 h (*p* < 0.01), cellular metabolic hydrogen recovery at 6 and 12 h (*p* < 0.03), and total metabolic hydrogen recovery across all time points (*p* < 0.01, 0.02, and 0.001; Supplementary Table S10).

### 3.3 Experiments 3 and 4—*In-vitro* fermentation profile

Effects of diets and additives are reported in Tables 6, 7, Supplementary Table S11, Figure 1B. Methane concentration in control cultures at 24 h was similar (*p* > 0.10). Culture pH and NH_3_ in control cultures decreased as the proportion of concentrate mix increased in the diet (Table 6).

**Table 6:** Effects of treatments combining sodium nitrate (N), sodium sulfate (S), 3-nitro-1-propionate (P), and bromochloromethane (B) on methane (CH_4_), pH and ammonia (NH_3_) after 6, 12, and 24 h of incubation with rumen microbes in an *in-vitro* mixed batch culture system fed varying forege:concentrate (HF = 70:30, MF = 50:50, and LF = 30:70) in experiments 3 and 4.

	**Diet** ^*****1*****^														
	**HF**				**MF**				**LF**					***p*** **<**		
**Item**	**Control**	**NS^*2*^**	**NSP^*3*^**	**NSPB^*4*^**	**Control**	**NS^*2*^**	**NSP^*3*^**	**NSPB^*4*^**	**Control**	**NS^*2*^**	**NSP^*3*^**	**NSPB^*4*^**	**SE**	**T^*5*^**	**D^*6*^**	**TxD^*7*^**
**6 Hr**																
CH_4_*, mM*	1.92	0.25	0.06	-0.03	1.92	0.26	0.11	-0.03	1.89	0.25	0.14	-0.03	0.10	0.001	0.970	1.00
pH	5.84	6.12	6.11	6.06	5.84	6.11	6.10	6.09	5.90	6.14	6.07	6.13	0.02	0.001	0.170	0.32
NH_3_*, mM*	2.91	9.16	6.53	6.30	4.27	9.11	7.41	5.82	5.83	8.96	7.21	9.29	1.51	0.001	0.110	0.51
**24 Hr**																
CH_4_*, mM*	4.50	0.23	0.16	-0.04	4.70	0.28	0.05	-0.04	4.33	0.94	0.06	-0.04	0.13	0.001	0.490	0.01
pH	5.42	5.72	5.66	5.65	5.33	5.59	5.59	5.56	5.29	5.50	5.53	5.47	0.01	0.001	0.001	0.01
NH_3_*, mM*	3.60	7.99	7.90	9.20	2.78	8.04	6.36	8.01	1.86	6.92	6.80	6.73	1.19	0.001	0.170	0.98

^*1*^HF, high forage; MF, medium forage; LF, low forage.

[-1pt]^*2*^NS = sodium nitrate + sodium sulfate (28 and 12 g/kg DM, respectively).

[-1pt]^*3*^NSP = NS + 3-nitro-1-propionate (2.0 g/kg DM).

[-1pt]^*4*^NSPB = NSP + bromochloromethane (0.3 g/kg DM).

[-1pt]^*5*^Treatment effect.

[-1pt]^*6*^Diet effect.

[-1pt]^*7*^Treatment and diet interaction.

**Table 7:** Effects of treatments combining sodium nitrate (N), sodium sulfate (S), 3-nitro-1-propionate (P), and bromochloromethane (B) on short chain fatty acids (SCFA) after 6, 12, and 24h of incubation with rumen microbes in an *in-vitro* mixed batch culture system fed varying forege:concentrate (HF = 70:30, MF = 50:50, and LF = 30:70) in experiments 3 and 4.

	**Diet** ^*****1*****^														
	**HF**				**MF**				**LF**					***p*** **<**		
**Item**	**Control**	**NS^*2*^**	**NSP^*3*^**	**NSPB^*4*^**	**Control**	**NS^*2*^**	**NSP^*3*^**	**NSPB^*4*^**	**Control**	**NS^*2*^**	**NSP^*3*^**	**NSPB^*4*^**	**SE**	**T^*5*^**	**D^*6*^**	**TxD^*7*^**
**6 Hr**																
Total SCFA*, mM*	68.23	69.31	65.28	36.84	60.94	65.39	57.58	35.49	55.17	56.07	61.22	43.48	7.35	0.001	0.350	0.64
Acetate (A)*, mol%*	68.49	73.20	71.69	64.66	67.71	73.92	76.41	65.68	66.29	73.06	78.05	74.93	5.28	0.010	0.230	0.31
Propionate (P)*, mol%*	21.70	18.74	20.04	28.99	20.00	17.96	17.82	24.33	18.34	18.38	15.76	18.98	2.38	0.010	0.020	0.53
Butyrate*, mol%*	7.03	5.64	5.03	2.18	9.65	5.61	4.30	6.32	11.78	5.84	4.09	3.94	2.79	0.001	0.290	0.38
Valerate*, mol%*	0.70	1.16	1.09	1.47	1.03	1.15	0.80	1.56	1.05	1.14	0.62	0.83	0.56	0.270	0.530	0.64
Isoacids*, mol%*	2.08	1.27	2.15	2.70	1.62	1.36	0.79	2.11	2.54	1.57	1.48	1.32	0.88	0.550	0.550	0.80
A:P	3.17	3.91	3.65	2.64	3.41	4.12	4.42	2.93	3.70	4.10	4.96	4.03	0.47	0.003	0.010	0.60
**24 Hr**																
Total SCFA*, mM*	89.72	89.70	79.80	82.97	95.35	92.08	82.57	86.95	99.50	94.20	81.22	89.38	7.79	0.010	0.260	0.99
Acetate (A)*, mol%*	61.41	60.69	56.35	51.48	55.65	55.49	52.57	47.76	48.12	50.87	48.83	36.76	5.16	0.001	0.001	0.80
Propionate (P)*, mol%*	21.33	25.46	25.98	27.91	20.30	27.38	28.20	27.69	23.41	26.95	28.91	28.82	1.48	0.001	0.090	0.64
Butyrate*, mol%*	15.10	10.88	13.26	17.71	20.88	14.33	15.81	20.77	25.80	19.44	19.49	30.73	3.13	0.001	0.001	0.46
Valerate*, mol%*	1.34	1.55	1.82	2.07	1.51	1.67	1.69	2.16	1.39	1.64	1.84	2.31	0.34	0.001	0.660	0.93
Isoacids*, mol%*	0.83	1.41	2.60	0.84	1.65	1.12	1.73	1.63	1.28	1.09	1.08	1.38	0.68	0.260	0.520	0.15
A:P	2.91	2.38	2.18	1.88	2.74	2.03	1.88	1.73	2.19	1.89	1.70	1.34	0.28	0.001	0.001	0.89

^*1*^HF, high forage; MF, medium forage; LF, low forage.

^*2*^NS = sodium nitrate + sodium sulfate (28 and 12 g/kg DM, respectively).

^*3*^NSP = NS + 3-nitro-1-propionate (2.0 g/kg DM).

^*4*^NSPB = NSP + bromochloromethane (0.3 g/kg DM).

^*5*^Treatment effect.

^*6*^Diet effect.

^*7*^Diet and treatment interaction.

At 24h, total SCFA in control cultures increased with an increase in the proportion of concentrate mix in the diet (Table 7). Acetate decreased and propionate and butyrate increased (*p* > 0.10) with an increase in dietary concentrate (Table 7). Molar proportions of valerate and isoacids were not affected (*p* > 0.10).

At 24h, there was an interaction between inclusion level of NaNO_3_ and diet on CH_4_ and culture pH (*p* < 0.01). All additive treatments decreased CH_4_ (*p* < 0.01) regardless of diet and increased (*p* < 0.01) pH and NH_3_ (Table 6). Methane decreased by 88, 97, and 99.5% on average by NS, NSP, and NSPB, respectively. Whereas the NSP treatment tended (*p* < 0.052) to increase H_2_, the NSPB increased (*p* < 0.028) H_2_ in the headspace of the culture bottles (Figure 1B).

There were significant effects (*p* < 0.01) of additives on fermentation profile. Diet had no effect (*p* > 0.10) on total SCFA. When averaged across diets, NSP decreased total SCFAs when compared with control (*p* < 0.007) and NS (*p* < 0.04); both NSP and NSPB decreased acetate when compared to the control and NS. All three additive treatments increased propionate when compared with the control, resulting in a decrease (*p* < 0.001) in the A:P ratio (Table 7). Cultures receiving the NSPB treatment had greater butyrate than those receiving the NS and NSP treatments. All treatments increased (*p* < 0.001) valerate while the NSP treatment increased (*p* < 0.05) isoacids in the HF diet compared to the control and NSPB treatment. The theoretical estimations of hexose metabolism, total metabolic hydrogen production, end-product, cellular, and total metabolic hydrogen recoveries were affected by the additive combinations (at least *p* < 0.01 across all of these estimations; Supplementary Table S11).

### 3.4 Amplicon sequencing data

#### 3.4.1 Species richness and evenness (alpha-diversity) in cultures

There was no significant effect (*p* > 0.05) of diet, additive treatment, or interaction thereof on alpha-diversity indices (Supplementary Figures S1A, S2A). For experiments 1 and 2, only the Chao1 diversity tended to differ (*p* < 0.052) by treatment (Supplementary Figure S1A).

#### 3.4.2 Community compositions (beta-diversity) in cultures

Among the control and highest inclusion levels of NaNO_3_, Na_2_SO_4_, 3NPA, and BCM in experiments 1 and 2, the treatment and diet individually altered the Bray-Curtis dissimilarity and weighted UniFrac distances (*p* < 0.05; Supplementary Figures S1B and S1C). The microbial communities between the control and NaNO_3_ were significantly different via the Bray-Curtis dissimilarity (*p* < 0.007) and weighted UniFrac distance (*p* < 0.001). On the other hand, the interaction term between treatment and diet changed (*p*_adj_ < 0.05) the Bray-Curtis dissimilarity and weighted UniFrac distances among the control and NS, NSP, NSPB treatments in experiments 3 and 4 (Supplementary Figure S2B). Microbial communities of NS (*p* < 0.03), NSP (*p* < 0.001), and NSPB (*p* < 0.04) treatments differed from that of the control via the Bray-Curtis dissimilarity, whereas only the NSP community differed (*p* < 0.003) from the control via the weighted UniFrac distance (Supplementary Figures S2B, C).

#### 3.4.3 Differential abundance analysis of microbial taxa

**Figure 2:**
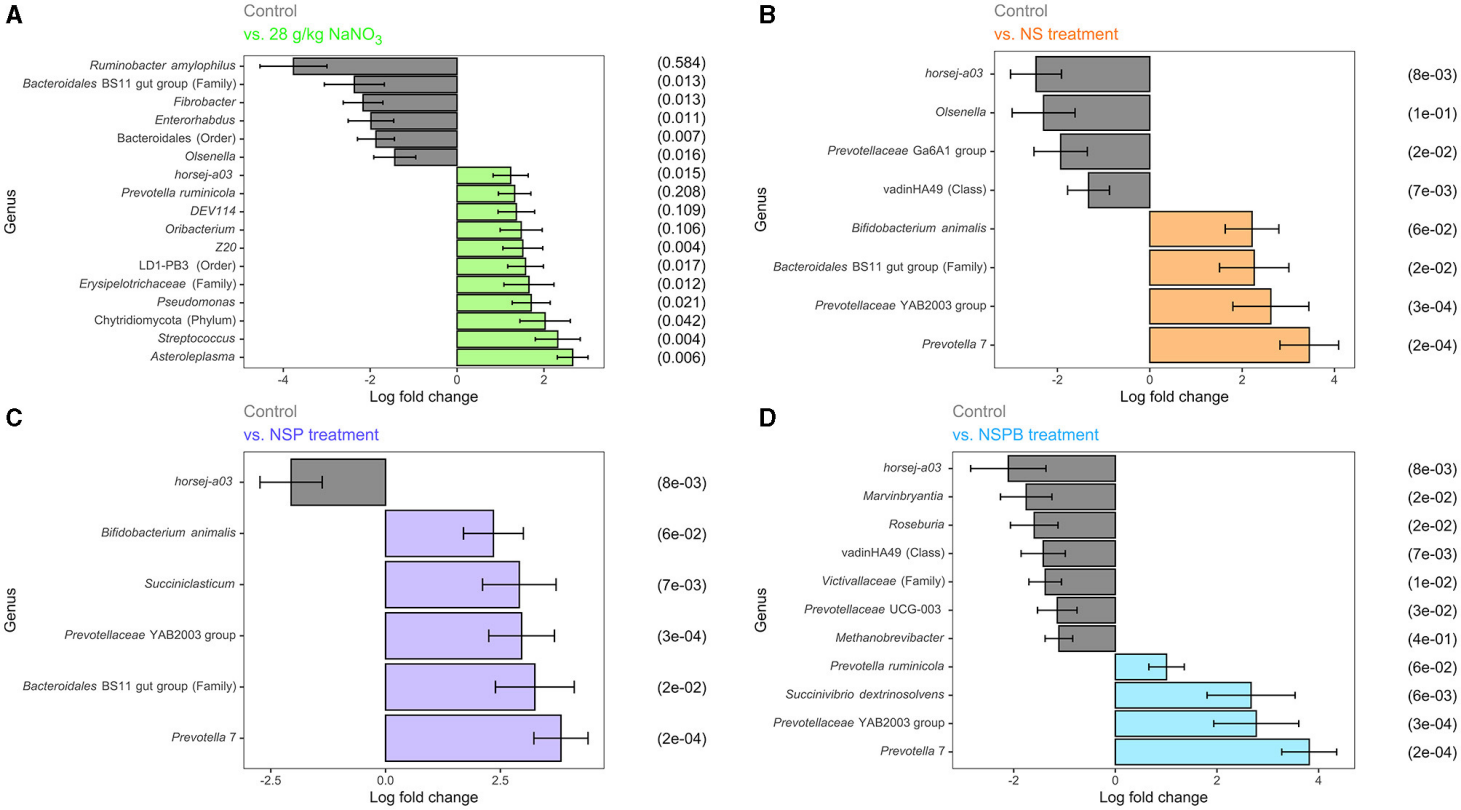
Effect of 28 g/kg DM sodium nitrate (NaNO_3_) and combination treatments with sodium nitrate + sodium sulfate (NS), NS + 3-nitro-1-propionate (NSP), and NSP + bromochloromethane (NSPB) on log fold change of microbial abundance on the species level after 24h of fermentation *in vitro*. Amplicon sequence variants (ASVs) were agglomerated at the lowest taxonomic level assigned. Taxonomy of these ASVs are annotated as follows. Genus and species are italicized; otherwise, the taxon of an unknown genus with its lowest available taxonomic rank (Family, Order, etc.) is indicated within parentheses. Using the ANCOMBC R package, log (ln) fold changes of differentially abundant taxa (p_adj_ < 0.05) between the control and: **(A)** 28 g/kg DM NaNO_3_; **(B)** combination of 28 g/kg DM NaNO_3_ and 12 g/kg DM Na_2_SO_4_ (NS); **(C)** combination of NS and 2.0 g/kg DM 3-nitro-1-propionate (NSP), and **(D)** combination of NSP and 0.30 g/kg DM bromochloromethane (NSPB). To the left of each subfigure is the relative abundance (%) of each taxon in the control group as a reference.

Differential abundance analysis of microbial taxa between the control and respective additive treatments were conducted on the genus-species level. For this analysis via ANCOMBC2, we were only interested in the treatment effect averaged across diets. Significant results (*p*_adj_ < 0.05) on the species level are shown for NaNO_3_, NS, NSP, and NSPB treatments in Figures 2A–D.

In experiments 1 and 2, the NaNO_3_ treatment affected the largest number of microbial taxa. On the species level, the addition of 28 g/kg of NaNO_3_ decreased (*p*_adj_ < 0.05) the abundances of *Ruminobacter amylophilus*, unassigned taxa belonging to the family *Bacteroidales* BS11 gut group, *Fibrobacter, Enterorhabdus*, unassigned taxa belonging to the order Bacteroidales, and *Olsenella* and increased (*p*_adj_ < 0.05) those of *Asteroleplasma, Streptococcus*, unassigned taxa belonging to the phylum Chytridiomycota, *Pseudomonas*, unassigned taxa belonging to the Family *Erysipelotrichaceae*, unassigned taxa belonging to the order LD-PB3, *Z20, Oribacterium, DEV114, Prevotella ruminicola*, and *horsej-a03*. On the other hand, the addition of 0.30 g/kg BCM increased (*p*_adj_ < 0.05) *Desulfovibrio* and *Megasphaera elsdenii*. The addition of Na_2_SO_4_ and 3NPA had no effect (*p*_adj_ > 0.10) on microbial taxa abundance.

In experiments 3 and 4, the addition of treatments from NS, NSP, and NSPB successively increased the number of differentially abundant microbial taxa compared to the control. The NS treatment decreased (p_adj_ < 0.05) the abundance of *horsej-a03, Olsenella, Prevotellaceae* Ga6A1 group, and unassigned taxa belonging to the class vadinHA49 and increased (p_adj_ < 0.05) *Prevotella* 7, *Prevotellaceae* YAB2003 group, unassigned taxa belonging to the family *Bacteroidales* BS11 gut group, and *Bifidobacterium animalis*. The NSP treatment decreased (p_adj_ < 0.05) *horsej-a03* and increased (p_adj_ < 0.05) *Prevotella* 7, unassigned taxa belonging to the family *Bacteroidales* BS11 gut group, *Prevotellaceae* YAB2003 group, *Succiniclasticum, Bifidobacterium animalis*. Lastly, the NSPB treatment decreased (p_adj_ < 0.05) the abundances of *horsej-a03, Marvinbryantia, Roseburia*, unassigned taxa belonging to the class vadinHA49, unassigned taxa belonging to the family *Victivallaceae, Prevotellaceae* UCG-003, and *Methanobrevibacter* and increased (p_adj_ < 0.05) *Prevotella* 7, *Prevotellaceae* YAB2003 group, *Succinivibrio dextrinosolvens*, and *Prevotella ruminicola*.

#### 3.4.4 Differential abundance analysis of predicted MetaCyc pathways

Differential abundance analyses of pathways predicted from the amplicon sequences were conducted between the control and each additive treatment via ANCOMBC2. In Experiments 1 and 2, 28 g/kg of NaNO_3_ decreased (p_adj_ < 0.05) the predicted pathway abundances of biotin biosynthesis II, L-glutamate degradation VIII to propanoate, TCA cycle VII of acetate-producers, and superpathway of polyamine biosynthesis I. Other additives did not affect (*p*_adj_ > 0.10) pathway abundances.

**Figure 3:**
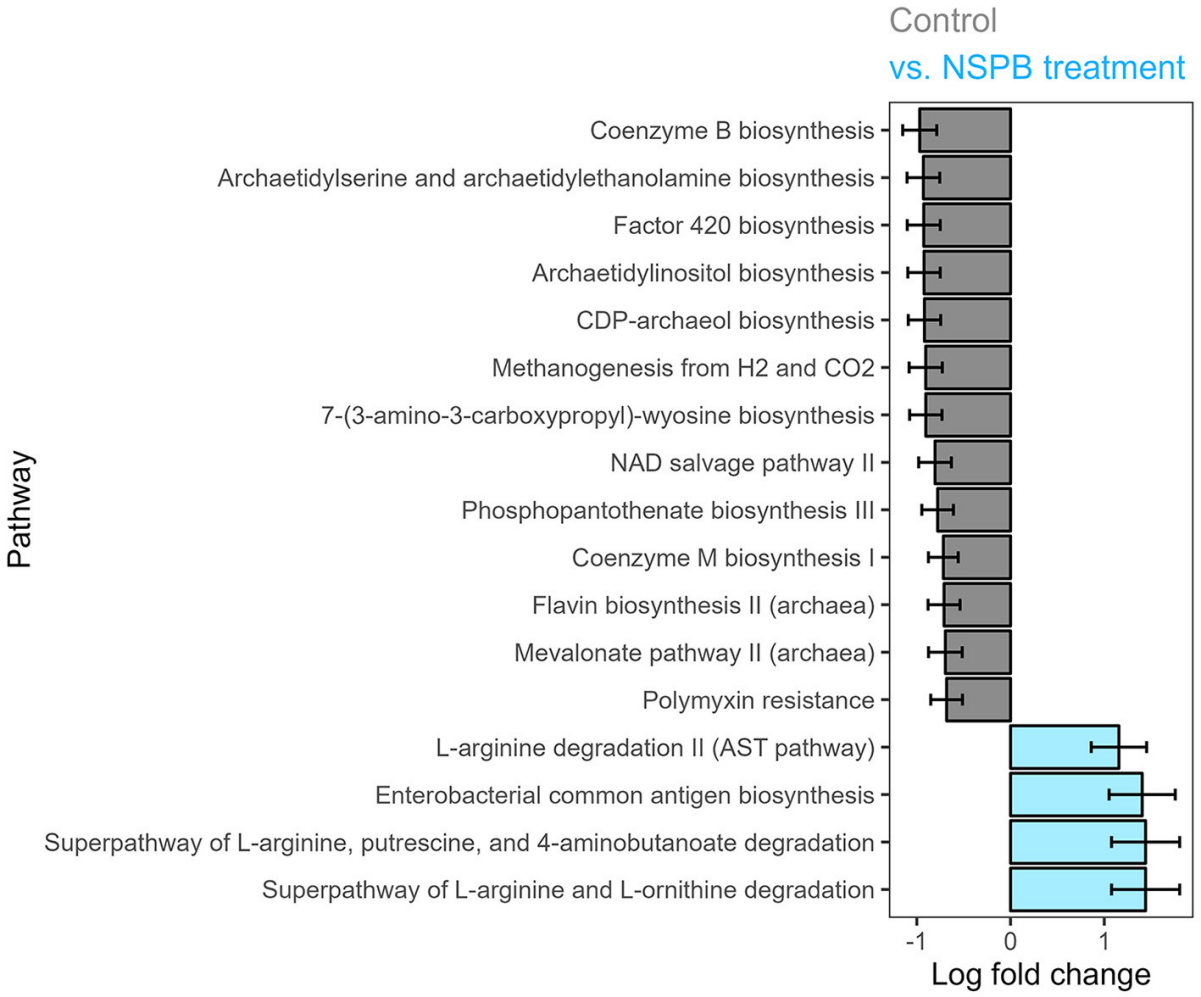
Effect of combination treatments with sodium nitrate (N), sodium sulfate (S), 3-nitro-1-propionate (P), and bromochloromethane (B) on log (ln) fold change of predicted MetaCyc pathway abundance on the species level after 24h of fermentation *in vitro*, calculated using the ANCOMBC package. Log fold changes of differentially abundant predicted pathways (p_adj_ < 0.05) between the control and combination of NaNO_3_, Na_2_SO_4_, 3-nitro-1-propionate, and bromochloromethane at 28, 12, 2.0, and 0.30 g/kd DM inclusion levels, respectively.

In experiments 3 and 4, only the NSP and NSPB treatments affected pathway abundances. In the NSP treatment, polymyxin resistance was decreased (*p*_adj_ < 0.05). The NSPB treatment decreased (*p*_adj_ < 0.05) coenzyme B biosynthesis, archaetidylserine and archaetidylethanolamine biosynthesis, factor 420 biosynthesis, archaetidylinositol biosynthesis, CDP-archaeol biosynthesis, methanogenesis from H_2_ and CO_2_, 7-(3-amino-3-carboxypropyl)-wyosine biosynthesis, NAD salvage pathway II, phosphopantothenate biosynthesis II (archaea), mevalonate pathway II (archaea), and polymyxin resistance while increasing superpathway of L-arginine and L-ornithine degradation, superpathway of L-arginine, putrescine, and 4-aminobutanoate degradation, enterobacterial common antigen biosynthesis, and L-arginine degradation II (AST pathway) (Figure 3).

## 4 Discussion

### 4.1 NaNO_3_, but not Na_2_SO_4_, inhibits *in-vitro* methanogenesis in a dose-dependent manner

In the anaerobic condition of the rumen, the economy of reduction and oxidation is managed through the microbial transaction of electrons as the currency. Plant carbohydrates are composed of polymers of hexoses and pentoses, which are liberated through hydrolysis and further metabolized by ruminal microbes through complete and incomplete glycolytic pathways (Hackmann et al., 2017). Glycolysis requires reduction of cofactors such as NAD^+^ to NADH that ultimately leads to the generation of ATP and GTP as a biological form of energy through substrate-level phosphorylation and electron transport phosphorylation (Ungerfeld, 2020). Through fermentation, microbial hydrogenases oxidize reduced cofactors, to maintain the redox recycling of cofactors, and generate H_2_, which is utilized by hydrogenotrophic biochemical reactions such as methanogenesis serving as a primary H_2_ sink (Ungerfeld, 2020).

Common thermodynamically favorable alternatives to methanogenesis are the reduction of nitrate (NO_3_^-^) and sulfate (SO_4_^2-^) (Thauer et al., 1977). Some plants accumulate NO_3_^-^ and SO_4_^2-^ due to abiotic stress from the environment (Giordano and Raven, 2014). In this study, NaNO_3_ was effective in inhibiting CH_4_ with almost 96% decline in CH_4_ when included at 28 g/kg DM level. Nitrate (NO_3_^-^) is well known for its anti-methanogenic potential due to its thermodynamic favorability of its reduction to nitrite (NO_2_^-^) and then to ammonia/ammonium (NH_3_/NH_4_^+^) with Gibbs free energy of -163.2 and -436.4 kJ/mol, respectively (Thauer et al., 1977). In comparison, the Gibbs free energy of hydrogenotrophic methanogenesis is -131 kJ/mol (Thauer et al., 1977). Additionally, NO_2_^-^ is toxic to the ruminant and ruminal microorganisms including methanogens (Takahashi and Young, 1991; Iwamoto et al., 2001), contributing to the anti-methanogenic effect.

Despite the favorable thermodynamics of sulfate as an electron acceptor, our data did not show any marked effects of SO_4_^2-^ on methanogenesis. Sulfate inclusion has been reported to be ineffective on ruminal methanogenesis below 20 mM (Ohashi et al., 1996). In the present study, the highest level (12 g/kg DM) of Na_2_SO_4_ equates to 2.8 mM. A similar inclusion level at 2.3 mM (Gupta et al., 2017) of Na_2_SO_4_ and 5 mM (Patra and Yu, 2014) of K_2_SO_4_ has been reported to be ineffective *in vitro*. A sulfate inclusion of 26 g/kg DM or larger has been shown to be effective in decreasing CH_4_ *in vivo* without the risk of polioencephalomalacia, particularly with dietary adaptation for the ruminal microbiota (van Zijderveld et al., 2010). Thus, had adaptation time for the sulfate inclusion been allowed, it may be possible for small inclusion levels such as mentioned above to show an effect *in vitro*.

Theoretically, NaNO_3_ owes its observed anti-methanogenic efficacy to two potential modes of action: first, it serves as a H_2_ sink during the reduction of NO_3_^-^ and NO_2_^-^; second, NO_2_^-^ accumulates and is toxic to methanogens. In the present study, it appears that the former was the major contributor of methanogenesis inhibition as the methanogen abundance did not significantly decrease were it for nitrite toxicity (Figure 2A).

The nitrate treatment enriched several other ASVs belonging to bacterial and fungal taxa that possess nitrate or ammonia assimilation capability (Figure 2A). *Pseudomonas* sp., enriched in the nitrate-treated cultures, likely possesses several genes essential to nitrate and nitrite metabolism: *narK* encoding nitrate/nitrite transporter, *nasA* encoding assimilatory nitrate reductase catalytic subunit, *nirB* and *nirD* encoding nitrite reductase (NADH) large and small subunits, as well as *ncd2* and *npd* encoding nitronate monooxygenase catalyzing the conversion of nitroalkane to acetaldehyde and nitrite (Nordberg et al., 2014). A fungal phylum Chytridiomycota was enriched in the nitrate treatment group compared to the control; Chytridiomycota fungi are capable of assimilating NO_3_^-^ as a nitrogen source (Digby et al., 2010). Though the present study does not provide clear distinction, the following two possibilities may explain the decrease in butyrate in the presence of nitrate-respiring microbes. First, the H_2_ partial pressure of nitrate-treated culture may have been low, wherein H_2_ is produced by NADH:ferredoxin oxidoreductase and hydrogenase, shunting NADH from the reduction of acetoacetyl-CoA to butyryl-CoA and thus decreasing butyrate (Macfarlane and Macfarlane, 2003). Another possibility is the conversion of butyrate to acetate, which also requires a low H_2_ partial pressure and produces reducing equivalents (McInerney et al., 2008; van Lingen et al., 2016).

In the nitrate-treated cultures, a notable increase in the abundance of ammonia-assimilating microbes was observed, among which was *Prevotella ruminicola*. The enrichment of *P. ruminicola* likely reflects the broader capacity of many ruminal microorganisms to reduce NO_3_^-^ and utilize NH_3_ as a nitrogen source, further enhancing its production (Kim et al., 2017). While *P. ruminicola* is generally recognized as a versatile ammonia assimilator and a ubiquitous fermenter in the rumen (Wang et al., 2015; Kim et al., 2017), the specific mechanisms driving the enrichment of this bacterium and possibly others remain unclear. It may be speculated, nonetheless, that the increased abundance exemplifies a high metabolic activity, perhaps warranted by the metabolic versatility, though abundance and metabolic activity do not always correlate (Stevenson and Weimer, 2007). The observed increase of metabolic hydrogen recovery in cells may reflect this phenomenon for the general microbial body in the nitrate-treated culture. Further, the metabolic hydrogen recovery was lower than that of the control and suggests incorporation of some reducing equivalents into pathways such as nitrate reduction other than those accounted for in the equation (i.e. CH_4_, H_2_, SCFAs).

### 4.2 3NPA may be a potent methanogenesis inhibitor that is metabolized rapidly

An additional purpose of the present study was to evaluate the effect of 3NPA on *in-vitro* methanogenesis. Naturally occurring in certain plants and fungi as a chemical defense against herbivores (Nishino et al., 2010; Francis et al., 2013; Torres-Guzman et al., 2021), 3NPA is reduced to β-alanine by ruminal microbes (Anderson et al., 1993), which is then metabolized in combination with pyruvate to malonate semialdehyde and then to acetyl-CoA and CO_2_ (Hayaishi et al., 1961; Nishino et al., 2010; Latham et al., 2016). However, 3NPA's mode of action in the rumen has not clearly been elucidated (Smith and Anderson, 2013) while its structural resemblance to 3-nitrooxypropanol (3NOP) may hint a possible enzymatic inhibition of methanogenesis similar to that of 3NOP [3NOP's mode of action described by Duin et al. (2016)].

In the present study, 3NPA inhibited methanogenesis by up to 54% when included at 2.0 g/kg DM level at 6h and 63% with 0.5 g/kg DM at 24h. The inhibitory effect of 3NPA was concomitant with a numerical increase in H_2_ concentration (Figure 1A) and a decrease in total SCFA concentration. Based on the results, the fermentation profiles of 3NPA-treated cultures were similar to those treated with BCM but to a lesser extent. Nevertheless, the mechanism of action for the anti-methanogenic effect of 3NPA remains unclear. Evaluating the effects of 3NPA on methanogenic enzymes, especially methyl-coenzyme M reductase, would elucidate whether or not the molecule serves as an enzymatic inhibitor in methanogenesis.

### 4.3 BCM inhibits *in-vitro* methanogenesis regardless of inclusion level

The present study also evaluated the effect of BCM on emphin-vitro fermentation. Belonging to one of the most potent categories of methane inhibitor, BCM is a CH_4_ analog and inhibits the cobamide-dependent methyl-transfer at the last step of methanogenesis (Wood et al., 1968). In contrast to NaNO_3_, an effective thermodynamic inhibitor, BCM resulted in nearly complete inhibition of methanogenesis irrespective of the inclusion levels. In BCM-treated cultures, H_2_ concentrations increased (*p* < 0.001) compared the control, NaNO_3_, Na_2_SO_4_, and 3NPA (Figure 1A). As expected, there was a significant increase in the molar proportion of propionate, as an internal sink of reducing equivalents, compared to the control throughout the incubation period. Additionally, at earlier incubation intervals (i.e. 6h), concentrations of NH_3_, as another internal H_2_ sink, in the BCM-treated cultures significantly increased despite the absence of any additional NaNO_3_.

When the ASVs were agglomerated to the genus and species levels based on their taxonomic assignment, *Desulfovibrio* and *Megasphaera elsdenii* were enriched in the BCM-treated culture than the control. Both bacterial species are related to internal H_2_ sinks in the rumen, sulfate and lactate/propionate, respectively; *Desulfovibrio* is a major sulfate-reducing bacterium in the rumen (Wu et al., 2021) while *M. elsdenii* is a lactate-fermenting and propionate-producing bacterium (Li et al., 2021), whose enrichment may suggest that H_2_ or reducing equivalents were partially redirected to sulfate reduction and lactate assimilation, respectively. However, it is clear that not all H_2_ was redistributed to internal H_2_ sinks, but rather, small amounts of H_2_ were released as gas evidenced by significantly increased (*p* < 0.001) H_2_ concentrations in culture treated with BCM (Figure 1A). The disturbance in the H_2_ concentration in culture likely contributed to the significantly declined total SCFA concentration (Janssen, 2010; Ungerfeld, 2020).

### 4.4 Combination of thermodynamic and enzymatic inhibitors additively inhibits methanogenesis

The NS and NSP treatments inhibited methanogenesis, on average, by 88 and 97%, respectively, similarly to the effect when NaNO_3_ was added as a single additive. Since the addition of Na_2_SO_4_ alone had no anti-methanogenic effect, it seems that the decline in CH_4_ in the NS treatment was solely due to NaNO_3_ with a possible antagonistic effect by Na_2_SO_4_ inclusion. 3NPA alone decreased CH_4_ by approximately 30% on average. Interestingly, when 3NPA was included with NaNO_3_, it further decreased CH_4_, and the mitigation effect was particularly greater in higher concentrate diets. The effect of BCM on methanogenesis was as expected, regardless of whether it was included alone or in combination with the other additives, i.e. the NSPB treatment. The effect of BCM was so drastic that cultures receiving BCM resulted in the buildup of H_2_ in the gaseous phase (*p* < 0.05; Figure 1B). However, it is intriguing that BCM alone did not seem to affect rumen NH_3_ at 24h, whereas NH_3_ increased substantially when the other additives were included with BCM (NSPB treatment), likely due to the inclusion of NaNO_3_.

We observed differences in the accounting of metabolic hydrogen recovery in cultures treated with potent inhibitors, NaNO_3_ and BCM alone and in combination (Supplementary Tables S7, S10, S11). At 24h, NaNO_3_ alone or in combination with Na_2_SO_4_ and 3NPA decreased the metabolic hydrogen recovery in fermentation end-products but increased that in cells. On the contrary, BCM alone decreased the latter. When combined, the effects of NaNO_3_ and BCM seem additive, in that the NSPB treatment decreased cellular metabolic hydrogen recovery and increased end-product and total metabolic hydrogen recovery compared to the NS and NSP treatment. Metabolic hydrogen recovery calculations suggest altered hydrogen metabolism directing hydrogen away from methanogenesis to fermentation products such as propionate and other hydrogenotrophic pathways such as nitrate reduction that are unaccounted for in the equations.

The drastic decline in *in-vitro* CH_4_ concentration was not concomitant with methanogen populations. In fact, several methanogen ASVs were enriched in the NS and NSP treatment. Notably, the relative abundance of methylotrophic methanogens belonging to the phylum Thermoplasmatota increased (*p* < 0.05) in the NS and NSP treatments compared to the control, but not in NaNO_3_, Na_2_SO_4_, and 3NPA individually (Supplementary Figure S3B). Because both NS and NSP treatments introduced competitive H_2_ (or reducing equivalent) sinks, methylotrophic methanogenesis may have partially occupied the niche space of methanogenesis under limited H_2_ availability; the methylotrophic pathway possesses a thermodynamic advantage over the hydrogenotrophic counterpart for H_2_ (or reducing equivalents) given the availability of methylated substrates (e.g. mono-, di-, and tri-methylamines) (Li et al., 2021).

In the NSPB treatment, the three following imputed pathways of L-arginine degradation were significantly enriched (Figure 3): (1) arginine succinyltransferase pathway; (2) superpathway of L-arginine, putrescine, and 4-aminobutanoate degradation; and 3) superpathway of L-arginine and L-ornithine degradation. If the imputation held true, these L-arginine catabolic pathways would lead to the production of succinate (Tabor and Tabor, 1985; Stalon et al., 1987; Kashiwagi et al., 1991). Succinate could then be utilized in propionate production by ruminal bacteria (Blackburn and Hungate, 1963). Among the propionate-producing bacteria in the present study, *P. ruminicola* depends on vitamin B_12_ for propionate production (Strobel, 1992). The B_12_ family of cofactors may have been more available in the absence of methanogenesis, which also relies on the same cofactors. Further, as with the NSPB treatment in this study, butyrate molar proportions have been reported to increase concomitantly with vitamin B_12_ concentration due to cobalt supplementation in a continuous culture (Tiffany et al., 2006). Concomitantly, the NSPB treatment decreased 12 imputed archaeal pathways (Figure 3), demonstrating specific suppression of the archaeal populations in the rumen, which are primarily composed of methanogens (Janssen and Kirs, 2008).

Bacteria of the family *Prevotellaceae* (*Prevotella* 7 and *Prevotellaceae* YAB2003 group) have been shown to promote propionate biosynthesis via succinate (Trautmann et al., 2022) and were enriched in all of the combination treatments (Figures 2B–D). ASVs identified as *Prevotella* 7 have > 97% sequence similarity to *P. albensis* on NCBI BLAST (Zhang et al., 2000). *P. albensis* appears to be capable of producing propionate from succinyl-CoA (Nordberg et al., 2014). Additionally, in the NSPB treatment, succinate-producing bacteria, *Succinivibrio dextrinosolvens* and *P. ruminicola*, were enriched. As mentioned above, the addition of NaNO_3_ alone increased the abundance of *P. ruminicola* (Figure 2A). Therefore, the enrichment of *Prevotellaceae* bacteria in the NaNO_3_, NS, NSP, and NSPB treatments may be attributed to the inclusion of NaNO_3_ (Abdelmoteleb et al., 2020; Hassan et al., 2021).

The enrichment of *Succiniclasticum* in the NSP treatment further attests to the increase in the conversion of succinate to propionate. *Succiniclasticum* is a unique bacterium that produces propionate from succinate as a sole energy source (van Gylswyk, 1995; Abbas et al., 2020). Also of note in the NSP treatment is the enrichment of *Denitrobacterium* at the genus level, as this bacterial genus is capable of metabolizing nitro-compounds such as reduction of NO_3_^-^ to NH_3_ and 3NPA to β-alanine (Anderson et al., 1993, 1996; Latham et al., 2016; Correa et al., 2017), which presumably was in response to the addition of NaNO_3_ and 3NPA in the treatment. As mentioned above, in the 3NPA-treated culture, we did not detect significant difference in the *Denitrobacterium* enrichment; however, this is not surprising given the early onset of anti-methanogenic effect from 3NPA observed at 6h in the present study, which seems to have subsided thereafter. The above pattern observed in the bacterial enrichment indicates the shuffling of H_2_ allocation, wherein succinate and ultimately propionate production seems to act as alternative sinks of reducing equivalents (Figure 1C). The estimated metabolic hydrogen recovery was lower than the control across the treatments, indicating the utilization of reducing equivalents in other reductive pathways.

The above results maintain that the thermodynamic and enzymatic modes of inhibition explored in this study acted independently of one another. The results herein provided may serve as a proof of concept, though limitations exist in this study, including the following: low cultural pH range, in which fiber degradation was likely impacted (Slyter, 1986), and so was methanogenesis (Sung et al., 2006); and use of one cannulated steer as the source of inoculum, which limits the statistical inference to this very steer. Thermodynamic inhibition by NaNO_3_ decreased CH_4_, as expected by the standard Gibbs free energy of -599.6 kJ/mol for the reduction of NO_3_^-^ to NH_3_ compared to that of -131.0 kJ/mol for methanogenesis (Thauer et al., 1977; Latham et al., 2016). Nitrate increased NH_3_ and propionate but decreased butyrate. On the other hand, enzymatic inhibition by BCM decreased CH_4_ via competitive inhibition as demonstrated by Wood et al. (1968), resulting in decreased acetate but increased propionate and gaseous H_2_. Presumably, NO_3_^-^ and BCM increased propionate via the succinate and acrylate pathways, respectively. Combining both NO_3_^-^ and BCM, the NSPB treatment decreased acetate but increased NH_3_, propionate, and valerate, as well as gaseous H_2_. The concomitant increase in propionate and enrichment of *Prevotella* spp. in the combination treatments may allude to a proliferation of atypical consortia of energy conserving microbiota (Hackmann et al., 2017). The accumulation of H_2_ can thermodynamically inhibit NADH oxidation, but electron acceptors (i.e., NO_3_^-^ reduction and succinate/propionate biosynthesis pathways by *Prevotella*), can recycle electrons from reduced ferredoxin; the redox state of NAD and ferredoxin serve as important drivers of ruminal fermentation processes (van Lingen et al., 2016).

### 4.5 Conclusion

Exploitation of thermodynamics for inhibition of methanogenesis follows the availability of alternative electron acceptors that are competitive to CH_4_ production. NO_3_^-^ demonstrated a dose-response inhibition of CH_4_. NO_3_^-^ as a competitive electron acceptor enriched ruminal microbes capable of nitrate/nitrite reduction as well as NH_3_/NH_4_^+^ assimilation. Multiple electron acceptors may inhibit methanogenesis given their thermodynamic competitiveness. Enzymatic inhibition, in contrast, was effective at all dose levels tested. Inhibition of methanogenesis, particularly with NaNO_3_ and BCM, upregulated internal H_2_ sinks including NH_3_ and propionate as supported by the enrichment of members of the family *Prevotellaceae* and *M. elsdenii*, respectively. The NSPB treatment decreased CH_4_ and the major ruminal methanogen *Methanobrevibacter*, but channeled reducing equivalents to NH_3_, propionate, and valerate. BCM also decreased CH_4_ but resulted in a build-up of gaseous H_2_. Both NSPB and BCM were associated with an increased propionate production. However, it seems that the accumulation of gaseous H_2_ by the enzymatic inhibition pose a detrimental impact on overall fermentation without an external H_2_ sink. Ruminal microbiome is known to adapt to shifts in dietary substrates via alteration of its profile and metabolic pathways. The results of the present study provide further evidence to the adaptability of the ruminal microbiome under suppression of methanogenesis.

## Data availability statement

The datasets analyzed for this study can be found in the NCBI SRA repository: BioProject ID PRJNA1020825; accession SAMN37529565-37529652.

## Ethics statement

The animal study was approved by NC State Institution of Animal Care and Use Committee. The study was conducted in accordance with the local legislation and institutional requirements.

## Author contributions

KT: Writing – review & editing, Writing – original draft, Visualization, Methodology, Investigation, Formal analysis, Data curation, Conceptualization. SC: Writing – review & editing, Validation, Resources, Formal analysis. KP: Writing – review & editing, Validation, Resources, Formal analysis. VF: Writing – review & editing, Supervision, Resources, Project administration, Methodology, Investigation, Funding acquisition, Formal analysis, Conceptualization.
