# Supplementary material for: Inhibiting methanogenesis by targeting thermodynamics and enzymatic reactions in mixed cultures of rumen microbes in vitro
Source: Front Microbiol. 2024 Aug 14;15:1322207. doi: 10.3389/fmicb.2024.1322207 (PMC11349738; doi:10.3389/fmicb.2024.1322207)
Supplement: Supplementary file 1 [file Data_Sheet_1.PDF]

Supplementary Material

1 SUPPLEMENTARY TABLES

Supplementary Table S1: Stock solution of additives and concentration

Additive	Amount, g	Solution	Amount, mL	Concentration, w/v %
$NaNO_3$	7.0	DI H_2O	25	28.0
Na_2SO_4	3.0	DI H_2O	25	12.0
3NPA	0.5	DI H_2O	25	2.0
BCM	0.3	MeOH	25	1.2

Abbreviations: 3NPA = 3-nitro-1-propionate; BCM = bromochloromethane; MeOH = methanol

Supplementary Table S2: Primer target genes and sequences used for Illumina MiSeq

Target gene	Primer sequence (5‘-3‘)
Universal 16S 515F	GTG CCA GCM GCC GCG GTA A
Universal 16S 806R	GAC TAC HVG GGT WTC TAA T
Archaea-specific 16S 516F	TGY CAG CCG CCG CGG TAA HAC CVG C
Archaea-specific 16S 915R	GTG CTC CCC CGC CAA TTC CT
Fungal ITS2 ITS3F	GCA TCG ATG AAG AAC GCA GC
Fungal ITS2 ITS4R	TCC TCC GCT TAT TGA TAT GC

Supplementary Table S3: Effect of sodium sulfate (Na_2SO_4) on methane (CH_4), pH, and ammonia (NH_3) after 6, 12, and 24h of incubation with rumen microbes in an *in-vitro* mixed batch culture system fed varying forege:concentrate (HF=70:30, MF=50:50, and LF=30:70) in experiment 1.

	Diet[1]															
	HF				MF				LF							
	Na_2SO_4, *g/kg DM*													p <		
Item	0	3	6	12	0	3	6	12	0	3	6	12	SE	D[2]	L[3]	DxL[4]
6 Hr																
CH_4, *mM*	1.33	1.32	1.32	1.21	1.19	1.29	1.40	1.34	1.48	1.52	1.44	1.45	0.11	0.010	0.78	0.67
pH	5.87	5.87	5.82	5.89	5.82	5.87	5.83	5.87	5.87	5.91	5.93	5.93	0.03	0.010	0.14	0.47
NH_3, *mM*	5.25	5.20	4.85	6.09	5.63	4.89	5.26	5.54	4.33	4.43	5.52	5.20	1.12	0.700	0.77	0.93
12 Hr																
CH_4, *mM*	1.98	2.12	2.07	1.86	2.28	1.96	1.90	2.13	1.91	1.52	0.91	1.94	0.50	0.040	0.31	0.44
pH	5.54	5.55	5.56	5.57	5.52	5.54	5.53	5.50	5.50	5.38	5.45	5.39	0.04	0.001	0.40	0.18
NH_3, *mM*	4.92	5.51	7.60	7.72	6.57	4.97	4.52	4.93	4.57	3.76	3.98	4.14	1.13	0.001	0.62	0.10
24 Hr																
CH_4, *mM*	1.66	1.46	1.62	1.58	1.81	2.15	2.27	2.39	2.49	2.53	2.04	2.30	0.78	0.001	0.75	0.02
pH	5.21	5.26	5.26	5.28	5.21	5.20	5.19	5.19	5.12	5.10	5.11	5.13	0.02	0.001	0.29	0.05
NH_3, *mM*	5.31	5.19	6.08	6.40	5.03	5.00	4.78	4.59	4.96	5.59	4.30	3.42	2.22	0.004	0.70	0.04

[1] HF = high forage; MF = medium forage; LF = low forage

[2] Diet effect

[3] Treatment level effect

[4] Diet and level interaction

Supplementary Table S4: Effect of sodium sulfate (Na_2SO_4) on short chain fatty acids (SCFA) after 6, 12, and 24h of incubation with rumen microbes in an *in-vitro* mixed batch culture system fed varying forege:concentrate (HF=70:30, MF=50:50, and LF=30:70) in experiment 1.

	Diet[1]															
	HF				MF				LF							
	Na_2SO_4, *g/kg DM*													p <		
Item	0	3	6	12	0	3	6	12	0	3	6	12	SE	D[2]	L[3]	DxL[4]
6 Hr																
Total SCFA, *mM*	83.87	86.76	83.34	83.94	84.65	81.09	83.05	80.64	75.95	70.16	80.41	77.93	8.59	0.270	0.97	0.98
Acetate (A), *mol%*	66.84	65.64	65.19	65.42	64.54	66.00	65.03	64.78	64.24	65.15	65.95	65.67	2.52	0.260	0.89	0.08
Propionate (P), *mol%*	24.99	25.66	26.06	25.88	25.72	24.84	25.74	25.59	24.83	24.49	23.25	23.43	1.90	0.004	0.98	0.42
Butyrate, *mol%*	6.99	7.20	7.32	7.25	8.26	7.82	7.88	8.11	9.30	8.83	9.03	9.01	0.59	0.001	0.95	0.98
Valerate, *mol%*	0.96	0.98	0.99	0.99	1.09	1.09	1.09	1.13	1.26	1.22	1.24	1.27	0.08	0.001	0.94	1.00
Isoacids, *mol%*	0.22	0.51	0.44	0.47	0.39	0.36	0.37	0.50	0.48	0.42	0.53	0.60	0.35	0.730	0.80	0.98
A:P	2.68	2.58	2.51	2.56	2.54	2.68	2.57	2.57	2.61	2.68	2.89	2.83	0.30	0.030	0.92	0.23
12 Hr																
Total SCFA, *mM*	110.67	108.64	112.74	106.37	100.14	106.79	112.21	112.49	103.61	108.80	103.00	109.12	7.53	0.410	0.39	0.22
Acetate (A), *mol%*	62.85	63.56	63.15	63.37	61.45	62.54	61.99	62.04	59.04	58.18	57.02	57.11	2.58	0.001	0.44	0.23
Propionate (P), *mol%*	27.01	26.22	26.17	26.03	26.74	26.04	26.30	26.06	26.47	26.79	27.08	26.78	3.20	0.200	0.55	0.65
Butyrate, *mol%*	8.69	8.71	9.10	9.05	10.35	9.93	10.11	10.38	12.88	13.66	14.41	14.50	0.96	0.001	0.47	0.84
Valerate, *mol%*	1.02	1.18	1.20	1.19	1.18	1.16	1.20	1.18	1.21	1.07	1.13	1.19	0.07	0.710	0.65	0.36
Isoacids, *mol%*	0.42	0.33	0.39	0.36	0.29	0.34	0.40	0.33	0.39	0.30	0.36	0.43	0.15	0.500	0.50	0.51
A:P	2.42	2.48	2.48	2.48	2.35	2.45	2.41	2.42	2.27	2.20	2.13	2.16	0.38	0.001	0.82	0.39
24 Hr																
Total SCFA, *mM*	134.72	145.14	141.06	147.78	138.63	134.90	135.64	138.86	129.56	141.56	141.58	138.95	4.21	0.140	0.10	0.32
Acetate (A), *mol%*	58.33	59.85	59.75	59.85	55.27	56.31	55.21	55.08	48.54	49.63	48.23	49.82	1.20	0.001	0.62	0.97
Propionate (P), *mol%*	26.84	26.91	26.30	26.74	27.69	25.95	25.75	25.42	26.93	27.13	26.47	26.22	1.69	0.240	0.04	0.27
Butyrate, *mol%*	13.34	11.71	12.39	11.97	15.61	16.23	17.35	17.86	22.70	21.12	23.18	22.18	1.93	0.001	0.70	0.89
Valerate, *mol%*	1.10	1.02	1.05	0.98	1.02	1.12	1.19	1.17	1.28	1.36	1.32	1.18	0.18	0.001	0.55	0.41
Isoacids, *mol%*	0.39	0.51	0.50	0.45	0.41	0.39	0.50	0.48	0.55	0.76	0.80	0.61	0.12	0.001	0.24	0.80
A:P	2.18	2.24	2.28	2.24	2.00	2.18	2.15	2.19	1.82	1.83	1.83	1.90	0.14	0.001	0.02	0.48

[1]HF = high forage; MF = medium forage; LF = low forage

[2]Diet effect

[3]Treatment level effect

[4]Diet and level interaction

Supplementary Table S5: Effect of sodium nitrate ($NaNO_3$) on the theoretical amount of hexose metabolized and production and recovery of metabolic hydrogen (2[H]) in an *in-vitro* mixed batch culture system fed varying forege:concentrate (HF=70:30, MF=50:50, and LF=30:70) in experiment 1.

	Diet[1]															
	HF				MF				LF							
	$NaNO_3$, *g/kg DM*													p <		
Item	0	7	14	28	0	7	14	28	0	7	14	28	SE	D[2]	L[3]	DxL[4]
6 Hr																
Hexose metabolized, *mmol*	1.36	1.42	1.30	0.82	1.38	1.34	1.20	0.97	1.19	1.18	1.00	0.84	0.21	0.300	0.010	0.95
Total 2[H] produced , *µmol*	3518.04	3554.41	3535.65	3446.91	3507.74	3525.33	3540.64	3559.15	3493.60	3488.48	3572.06	3610.03	50.20	0.640	0.640	0.27
2[H] recovered in end-products*	1591.74	1287.05	1230.07	1264.14	1570.25	1347.31	1257.89	1179.55	1737.95	1598.65	1265.60	1147.81	83.02	0.160	0.001	0.18
2[H] recovered in cells*	339.06	339.46	343.73	352.18	334.60	339.95	341.32	345.56	331.34	334.32	337.87	341.68	2.88	0.001	0.001	0.75
Total 2[H] recovered, *%*	54.90	45.78	44.52	47.33	54.32	47.76	45.06	42.74	59.10	55.28	44.78	41.16	3.11	0.460	0.001	0.23
12 Hr																
Hexose metabolized, *mmol*	1.82	1.85	1.83	1.52	1.67	1.79	1.85	1.43	1.77	1.70	1.92	1.52	0.17	0.280	0.001	0.36
Total 2[H] produced , *µmol*	3485.64	3517.37	3509.16	3532.67	3496.58	3480.56	3513.37	3532.57	3512.00	3522.45	3507.90	3525.58	64.28	0.660	0.160	0.68
2[H] recovered in end-products*	1676.64	1556.69	1427.51	1186.89	1796.42	1640.95	1522.09	1225.19	1773.47	1715.73	1663.41	1254.01	36.03	0.001	0.001	0.08
2[H] recovered in cells*	333.55	332.09	336.50	345.23	328.22	331.44	330.16	341.95	320.73	321.25	319.93	340.36	4.50	0.001	0.001	0.05
Total 2[H] recovered, *%*	57.72	53.72	50.29	43.39	60.76	56.78	52.72	44.38	59.69	57.86	56.59	45.26	1.88	0.001	0.001	0.07
24 Hr																
Hexose metabolized, *mmol*	2.31	2.51	2.49	2.21	2.42	2.47	2.42	2.17	2.39	2.47	2.59	2.35	0.08	0.260	0.001	0.59

Total 2[H] produced , *µmol*	3510.07	3526.34	3493.37	3466.95	3505.95	3530.59	3516.14	3469.81	3540.76	3559.58	3542.07	3482.77	42.28	0.010	0.001	0.94
2[H] recovered in end-products*	1690.62	1630.33	1585.08	1416.75	1773.36	1740.00	1684.82	1520.12	1986.40	1907.23	1830.88	1688.55	50.95	0.001	0.001	0.98
2[H] recovered in cells*	319.69	320.97	324.04	334.49	313.63	311.98	313.85	325.23	295.29	294.21	296.96	308.45	6.49	0.001	0.001	1.00
Total 2[H] recovered, %	57.28	55.34	54.66	50.54	59.53	58.11	56.85	53.20	64.42	61.86	60.06	57.32	1.00	0.001	0.001	0.98

[1]HF = high forage; MF = medium forage; LF = low forage
[2]Diet effect
[3]Treatment level effect
[4]Diet and level interaction
*Unit in *µmol / mmol hexose metabolized*

Supplementary Table S6: Effect of sodium sulfate (Na_2SO_4) on the theoretical amount of hexose metabolized and production and recovery of metabolic hydrogen (2[H]) in an *in-vitro* mixed batch culture system fed varying forege:concentrate (HF=70:30, MF=50:50, and LF=30:70) in experiment 1.

	Diet[1]															
	HF				MF				LF							
	Na_2SO_4, *g/kg DM*													p <		
Item	0	3	6	12	0	3	6	12	0	3	6	12	SE	D[2]	L[3]	DxL[4]
6 Hr																
Hexose metabolized, *mmol*	1.36	1.40	1.35	1.36	1.38	1.32	1.35	1.32	1.25	1.15	1.32	1.28	0.14	0.400	0.97	0.98
Total 2[H] produced , *μmol*	3518.04	3505.09	3498.37	3501.51	3507.74	3522.05	3505.75	3508.60	3526.21	3531.14	3553.89	3549.36	35.40	0.002	0.99	0.49
2[H] recovered in end-products*	1591.74	1557.07	1583.74	1551.47	1570.25	1564.28	1629.66	1608.35	1672.19	1660.97	1573.57	1592.92	65.83	0.300	0.92	0.73
2[H] recovered in cells*	339.06	338.18	337.81	338.03	334.60	335.98	335.79	334.92	330.93	332.46	331.75	331.69	2.14	0.001	0.97	0.99
Total 2[H] recovered, *%*	54.90	54.10	54.94	54.00	54.32	53.96	56.10	55.42	56.83	56.48	53.67	54.26	2.32	0.750	0.94	0.74
12 Hr																
Hexose metabolized, *mmol*	1.82	1.79	1.86	1.75	1.67	1.78	1.87	1.88	1.77	1.87	1.78	1.89	0.14	0.830	0.34	0.33
Total 2[H] produced , *μmol*	3485.64	3499.29	3501.41	3503.89	3496.58	3508.07	3503.56	3509.33	3512.00	3513.23	3510.13	3514.90	61.22	0.080	0.47	0.97
2[H] recovered in end-products*	1676.64	1686.01	1676.05	1657.61	1796.42	1683.65	1681.31	1709.46	1773.47	1715.06	1657.63	1801.84	67.08	0.120	0.16	0.42
2[H] recovered in cells*	333.55	333.00	331.73	331.92	328.22	329.39	328.70	328.01	320.73	318.93	316.62	316.22	2.94	0.001	0.44	0.92
Total 2[H] recovered, *%*	57.72	57.73	57.38	56.81	60.76	57.42	57.42	58.10	59.69	57.96	56.30	60.30	2.80	0.300	0.08	0.35
24 Hr																
Hexose metabolized, *mmol*	2.31	2.44	2.39	2.49	2.42	2.37	2.40	2.47	2.39	2.59	2.63	2.56	0.08	0.020	0.17	0.58

Total 2[H] produced , *μmol*	3510.07	3502.30	3515.61	3507.16	3505.95	3536.86	3542.92	3550.61	3540.76	3530.44	3548.43	3552.40	32.46	0.001	0.13	0.37
2[H] recovered in end-products*	1690.62	1614.01	1631.36	1626.33	1773.36	1777.90	1812.21	1812.06	1986.40	1945.61	1899.55	1901.93	72.15	0.001	0.28	0.20
2[H] recovered in cells*	319.69	324.49	322.46	323.88	313.63	311.73	308.65	307.37	295.29	298.83	294.01	296.68	5.02	0.001	0.72	0.85
Total 2[H] recovered, *%*	57.28	55.35	55.57	55.60	59.53	59.07	59.84	59.69	64.42	63.55	61.78	61.87	1.41	0.001	0.05	0.18

[1] HF = high forage; MF = medium forage; LF = low forage

[2] Diet effect

[3] Treatment level effect

[4] Diet and level interaction

* Unit in *μmol / mmol hexose metabolized*

Supplementary Table S7: Effect of 3-nitro-1-propionate (3NPA) on methane (CH_4), pH and ammonia (NH_3) after 6, 12, and 24h of incubation with rumen microbes in an *in-vitro* mixed batch culture system fed varying forege:concentrate (HF=70:30, MF=50:50, and LF=30:70) in experiment 2.

	Diet[1]															
	HF				MF				LF							
	3NPA, *g/kg DM*													p <		
Item	0	0.5	1	2	0	0.5	1	2	0	0.5	1	2	SE	D[2]	L[3]	DxL[4]
6 Hr																
CH_4, *mM*	1.33	0.93	1.01	0.61	1.19	1.20	1.09	0.74	1.48	1.10	1.37	0.88	0.29	0.010	0.001	0.370
pH	5.87	5.87	5.85	5.79	5.82	5.83	5.85	5.85	5.87	5.87	5.83	5.90	0.02	0.240	0.910	0.120
NH_3, *mM*	5.25	5.53	6.22	6.46	5.63	7.34	7.42	7.40	5.50	6.38	6.43	6.67	1.02	0.140	0.200	0.980
12 Hr																
CH_4, *mM*	1.98	1.28	1.37	1.38	2.28	1.84	1.51	1.41	1.91	1.65	1.82	1.57	0.54	0.080	0.001	0.360
pH	5.54	5.43	5.44	5.46	5.52	5.40	5.40	5.41	5.49	5.32	5.36	5.39	0.03	0.001	0.001	0.720
NH_3, *mM*	4.92	5.97	5.64	5.54	6.57	5.41	11.69	5.73	4.57	3.88	1.78	4.20	2.34	0.050	0.860	0.380
24 Hr																
CH_4, *mM*	1.66	0.60	1.93	1.46	1.81	2.05	1.81	0.97	2.49	1.81	2.07	1.61	0.74	0.003	0.002	0.005
pH	5.21	5.33	5.27	5.28	5.21	5.20	5.22	5.23	5.12	5.11	5.13	5.13	0.02	0.001	0.090	0.030
NH_3, *mM*	5.31	5.75	5.71	6.57	5.03	5.82	5.79	5.17	4.96	2.51	3.74	2.75	1.17	0.001	0.920	0.380

[1] HF = high forage; MF = medium forage; LF = low forage

[2] Diet effect

[3] Treatment level effect

[4] Diet and level interaction

Supplementary Table S8: Effect of 3-nitro-1-propionate (3NPA) on short chain fatty acids (SCFA) after 6, 12, and 24h of incubation with rumen microbes in an *in-vitro* mixed batch culture system fed varying forege:concentrate (HF=70:30, MF=50:50, and LF=30:70) in experiment 2.

	Diet[1]															
	HF				MF				LF							
	3NPA, *g/kg DM*													p <		
Item	0	0.5	1	2	0	0.5	1	2	0	0.5	1	2	SE	D[2]	L[3]	DxL[4]
6 Hr																
Total SCFA, *mM*	83.87	87.19	79.77	84.30	84.65	70.44	75.13	66.76	79.13	74.80	73.60	66.82	7.39	0.090	0.450	0.83
Acetate (A), *mol%*	66.84	66.25	65.99	63.94	64.54	64.95	64.96	64.46	62.44	66.46	66.55	66.40	1.06	0.170	0.150	0.03
Propionate (P), *mol%*	24.99	26.29	26.60	28.18	25.72	26.73	26.42	27.44	24.38	24.91	24.82	24.56	2.37	0.004	0.130	0.70
Butyrate, *mol%*	6.99	6.33	6.35	6.70	8.26	7.09	7.11	6.97	10.84	7.54	7.57	7.84	1.46	0.002	0.020	0.56
Valerate, *mol%*	0.96	0.84	0.86	0.90	1.09	1.15	1.15	1.10	1.45	0.97	0.98	1.01	0.18	0.001	0.070	0.12
Isoacids, *mol%*	0.22	0.30	0.21	0.27	0.39	0.07	0.36	0.04	0.76	0.12	0.08	0.20	0.22	0.900	0.380	0.64
A:P	2.68	2.53	2.49	2.28	2.54	2.45	2.51	2.39	2.57	2.74	2.71	2.74	0.26	0.010	0.380	0.23
12 Hr																
Total SCFA, *mM*	110.67	118.99	105.26	117.27	100.14	110.83	109.95	107.00	103.60	105.21	105.23	98.66	6.41	0.020	0.330	0.37
Acetate (A), *mol%*	62.85	59.75	60.25	60.17	61.45	59.45	59.80	58.87	59.04	56.81	58.63	58.14	0.90	0.001	0.010	0.82
Propionate (P), *mol%*	27.01	29.05	28.35	28.07	26.74	26.91	27.78	27.95	26.47	26.19	24.39	26.90	2.16	0.004	0.520	0.34
Butyrate, *mol%*	8.69	9.84	10.12	10.20	10.35	12.23	11.09	11.86	12.88	15.52	15.64	13.66	1.57	0.001	0.001	0.15
Valerate, *mol%*	1.02	1.13	1.19	1.29	1.18	1.18	1.15	1.23	1.21	1.16	1.14	1.10	0.16	0.850	0.740	0.39
Isoacids, *mol%*	0.42	0.22	0.09	0.26	0.29	0.23	0.17	0.10	0.39	0.32	0.19	0.19	0.08	0.410	0.020	0.79
A:P	2.42	2.06	2.13	2.15	2.35	2.24	2.16	2.11	2.27	2.17	2.41	2.20	0.21	0.610	0.100	0.42
24 Hr																
Total SCFA, *mM*	134.72	135.27	136.12	133.39	138.63	128.57	122.25	117.55	129.56	129.71	126.88	125.87	3.72	0.010	0.040	0.15
Acetate (A), *mol%*	58.33	56.67	58.15	56.72	55.27	55.00	55.12	53.83	48.54	51.40	52.84	51.15	0.95	0.001	0.240	0.14
Propionate (P), *mol%*	26.84	26.85	26.21	27.23	27.69	25.60	25.52	26.76	26.93	25.70	25.56	25.87	2.08	0.140	0.020	0.54
Butyrate, *mol%*	13.34	14.63	13.88	14.25	15.61	17.77	17.74	17.84	22.70	21.39	20.04	21.24	2.12	0.001	0.690	0.27
Valerate, *mol%*	1.10	1.38	1.31	1.38	1.02	1.23	1.26	1.28	1.28	1.14	1.15	1.33	0.24	0.140	0.010	0.06
Isoacids, *mol%*	0.39	0.48	0.45	0.43	0.41	0.40	0.35	0.29	0.55	0.38	0.40	0.41	0.08	0.390	0.730	0.78
A:P	2.18	2.15	2.24	2.12	2.00	2.16	2.17	2.02	1.82	2.00	2.08	1.99	0.16	0.001	0.010	0.42

[1]HF = high forage; MF = medium forage; LF = low forage

[2]Diet effect

[3]Treatment level effect

[4]Diet and level interaction

Supplementary Table S9: Effect of 3-nitro-1-propionate (3NPA) on the theoretical amount of hexose metabolized and production and recovery of metabolic hydrogen (2[H]) in an *in-vitro* mixed batch culture system fed varying forege:concentrate (HF=70:30, MF=50:50, and LF=30:70) in experiment 2.

	Diet[1]															
	HF				MF				LF					p <		
	3NPA, *g/kg DM*															
Item	0	0.5	1	2	0	0.5	1	2	0	0.5	1	2	SE	D[2]	L[3]	DxL[4]
6 Hr																
Hexose metabolized, *mmol*	1.36	1.40	1.28	1.36	1.38	1.15	1.22	1.08	1.33	1.22	1.20	1.09	0.12	0.170	0.390	0.86
Total 2[H] produced , *µmol*	3518.04	3491.89	3486.46	3457.28	3507.74	3483.79	3487.89	3470.22	3538.10	3521.44	3523.55	3528.04	47.54	0.002	0.080	0.81
2[H] recovered in end-products*	1591.74	1467.71	1516.69	1490.96	1570.25	1624.49	1575.71	1533.20	1727.75	1521.28	1585.63	1488.27	36.38	0.010	0.001	0.01
2[H] recovered in cells*	339.06	341.55	341.44	340.20	334.60	338.18	338.05	338.84	325.40	337.36	337.26	336.39	5.18	0.003	0.020	0.53
Total 2[H] recovered, *%*	54.90	51.81	53.29	52.95	54.32	56.36	54.91	53.96	58.44	52.82	54.59	51.72	1.10	0.080	0.020	0.04
12 Hr																
Hexose metabolized, *mmol*	1.82	1.98	1.76	1.95	1.67	1.88	1.85	1.82	1.77	1.84	1.84	1.70	0.13	0.240	0.170	0.38
Total 2[H] produced , *µmol*	3485.64	3454.53	3468.33	3471.41	3496.58	3502.54	3483.25	3483.32	3512.00	3529.82	3562.01	3509.88	43.59	0.001	0.690	0.29
2[H] recovered in end-products*	1676.64	1667.97	1682.67	1668.76	1796.42	1731.92	1697.24	1713.82	1773.47	1765.61	1727.91	1746.89	47.49	0.010	0.370	0.82
2[H] recovered in cells*	333.55	329.84	328.93	328.31	328.22	322.77	326.07	323.66	320.73	313.53	313.35	318.98	5.00	0.001	0.001	0.20
Total 2[H] recovered, *%*	57.72	57.82	57.99	57.52	60.76	58.66	58.09	58.50	59.69	58.90	57.31	58.88	1.13	0.280	0.390	0.84
24 Hr																
Hexose metabolized, *mmol*	2.31	2.35	2.34	2.31	2.42	2.29	2.18	2.10	2.39	2.37	2.30	2.30	0.07	0.110	0.080	0.33

Total 2[H] produced , *μmol*	3510.07	3510.35	3519.99	3502.29	3505.95	3546.75	3547.79	3527.06	3540.76	3559.50	3557.04	3553.76	39.75	0.001	0.030	0.49
2[H] recovered in end-products*	1690.62	1599.83	1719.30	1707.92	1773.36	1792.70	1766.59	1711.85	1986.40	1829.08	1837.22	1826.42	74.07	0.001	0.050	0.03
2[H] recovered in cells*	319.69	315.42	317.58	316.45	313.63	307.51	307.51	307.38	295.29	298.78	301.90	298.57	5.92	0.001	0.580	0.24
Total 2[H] recovered, *%*	57.28	54.59	57.86	57.80	59.53	59.20	58.43	57.23	64.42	59.76	60.13	59.78	1.43	0.001	0.020	0.05

[1]HF = high forage; MF = medium forage; LF = low forage
[2]Diet effect
[3]Treatment level effect
[4]Diet and level interaction
*Unit in *μmol / mmol hexose metabolized*

Supplementary Table S10: Effect of bromochloromethane (BCM) on the theoretical amount of hexose metabolized and production and recovery of metabolic hydrogen (2[H]) in an *in-vitro* mixed batch culture system fed varying forege:concentrate (HF=70:30, MF=50:50, and LF=30:70) in experiment 2.

	Diet[1]															
	HF				MF				LF							
	BCM, *g/kg DM*													p <		
Item	0	0.075	0.15	0.3	0	0.075	0.15	0.3	0	0.075	0.15	0.3	SE	D[2]	L[3]	DxL[4]
6 Hr																
Hexose metabolized, *mmol*	1.36	1.32	1.33	1.33	1.38	1.25	1.07	0.98	1.31	1.19	1.25	1.12	0.12	0.130	0.250	0.690
Total 2[H] produced , *μmol*	3518.04	3424.70	3434.38	3438.88	3507.74	3411.40	3415.18	3416.67	3527.74	3455.01	3445.99	3457.47	37.45	0.010	0.001	0.980
2[H] recovered in end-products*	1591.74	1444.48	1438.13	1432.58	1570.25	1565.36	1516.12	1497.32	1711.68	1514.39	1449.50	1413.26	33.79	0.030	0.001	0.040
2[H] recovered in cells*	339.06	339.99	338.39	338.01	334.60	331.48	334.86	336.25	325.34	328.22	334.83	336.49	5.39	0.001	0.200	0.280
Total 2[H] recovered, *%*	54.90	52.10	51.73	51.49	54.32	55.66	54.22	53.68	57.99	53.37	51.72	50.63	1.32	0.070	0.004	0.170
12 Hr																
Hexose metabolized, *mmol*	1.82	1.80	1.83	2.02	1.67	1.57	1.68	1.72	1.77	1.70	1.59	1.63	0.10	0.001	0.170	0.090
Total 2[H] produced , *μmol*	3485.64	3436.40	3426.80	3414.53	3496.58	3399.96	3418.95	3431.08	3512.00	3466.77	3469.89	3465.55	37.87	0.003	0.001	0.680
2[H] recovered in end-products*	1676.64	1527.33	1563.22	1589.44	1796.42	1636.26	1634.59	1632.08	1773.47	1664.71	1665.27	1671.33	21.70	0.001	0.001	0.490
2[H] recovered in cells*	333.55	329.61	328.07	327.79	328.22	326.53	322.96	320.94	320.73	311.47	310.79	311.01	4.99	0.001	0.001	0.570
Total 2[H] recovered, *%*	57.72	54.03	55.19	56.17	60.76	57.74	57.27	56.93	59.69	57.00	56.95	57.20	0.97	0.001	0.001	0.610
24 Hr																
Hexose metabolized, *mmol*	2.31	1.94	2.02	2.28	2.42	2.13	2.16	2.16	2.39	2.01	2.28	2.22	0.17	0.540	0.010	0.770

Total 2[H] produced , *μmol*	3510.07	3472.01	3454.14	3451.43	3505.95	3469.91	3471.47	3458.76	3540.76	3465.86	3490.70	3469.68	31.38	0.010	0.001	0.180
2[H] recovered in end-products*	1690.62	1691.57	1709.88	1719.19	1773.36	1757.78	1779.34	1804.98	1986.40	1784.16	1803.71	1832.23	68.22	0.001	0.010	0.001
2[H] recovered in cells*	319.69	308.17	309.57	309.16	313.63	302.29	299.95	300.00	295.29	300.10	294.20	295.42	10.19	0.001	0.020	0.250
Total 2[H] recovered, *%*	57.28	57.59	58.46	58.77	59.53	59.36	59.88	60.85	64.42	60.14	60.08	61.31	1.21	0.001	0.040	0.002

[1]HF = high forage; MF = medium forage; LF = low forage
[2]Diet effect
[3]Treatment level effect
[4]Diet and level interaction
*Unit in *μmol / mmol hexose metabolized*

Supplementary Table S11: Effects of treatments combining sodium nitrate (N), sodium sulfate (S), 3-nitro-1-propionate (P), and bromochloromethane (B) on the theoretical amount of hexose metabolized and production and recovery of metabolic hydrogen (2[H]) in an *in-vitro* mixed batch culture system fed varying forege:concentrate (HF=70:30, MF=50:50, and LF=30:70) in experiments 3 and 4.

	Diet[1]															
	HF				MF				LF					p <		
Item	Control	NS[2]	NSP[3]	NSPB[4]	Control	NS[2]	NSP[3]	NSPB[4]	Control	NS[2]	NSP[3]	NSPB[4]	SE	T[5]	D[6]	TxD[7]
6 Hr																
Hexose metabolized, *mmol*	1.08	1.10	1.02	0.56	0.99	1.03	0.90	0.55	0.91	0.89	0.95	0.67	0.11	0.001	0.440	0.69
Total 2[H] produced , *μmol*	3576.06	3622.51	3593.05	3394.66	3614.57	3637.24	3643.51	3510.27	3647.41	3629.81	3682.10	3618.43	52.73	0.010	0.030	0.53
2[H] recovered in end-products*	1656.46	1070.27	1069.42	1354.87	1709.01	1049.79	938.82	1258.47	1748.36	1083.48	856.47	941.97	182.44	0.001	0.230	0.30
2[H] recovered in cells*	339.27	342.96	344.51	352.81	330.33	342.76	348.20	340.48	323.76	341.85	349.54	349.64	10.36	0.001	0.380	0.29
Total 2[H] recovered, %	55.84	39.01	39.41	51.60	56.44	38.28	35.32	45.95	56.85	39.33	32.75	35.76	5.69	0.001	0.150	0.36
24 Hr																
Hexose metabolized, *mmol*	1.55	1.49	1.35	1.47	1.72	1.59	1.43	1.58	1.87	1.70	1.46	1.75	0.11	0.001	0.001	0.88
Total 2[H] produced , *μmol*	3607.99	3513.30	3505.44	3497.08	3638.67	3494.15	3483.20	3507.88	3608.40	3523.31	3488.20	3528.43	20.55	0.001	0.850	0.46
2[H] recovered in end-products*	2181.86	1464.42	1558.31	1662.03	2246.27	1618.31	1646.45	1735.83	2303.49	1812.00	1738.87	1934.83	151.34	0.001	0.003	0.89
2[H] recovered in cells*	314.05	325.07	317.00	305.96	298.29	315.04	310.81	297.23	287.41	301.69	301.79	275.26	8.48	0.001	0.001	0.57
Total 2[H] recovered, %	69.20	50.94	53.52	56.32	69.93	55.34	56.23	57.96	72.00	60.00	58.44	62.71	4.34	0.001	0.010	0.94

[1]HF = high forage; MF = medium forage; LF = low forage
[2]NS = sodium nitrate + sodium sulfate (28 and 12 g/kg DM, respectively)
[3]NSP = NS + 3-nitro-1-propionate (2.0 g/kg DM)
[4]NSPB = NSP + bromochloromethane (0.3 g/kg DM)
[5]Treatment effect
[6]Diet effect
[7]Treatment and diet interaction
[*]Unit in *μmol / mmol hexose metabolized*

2 SUPPLEMENTARY FIGURES

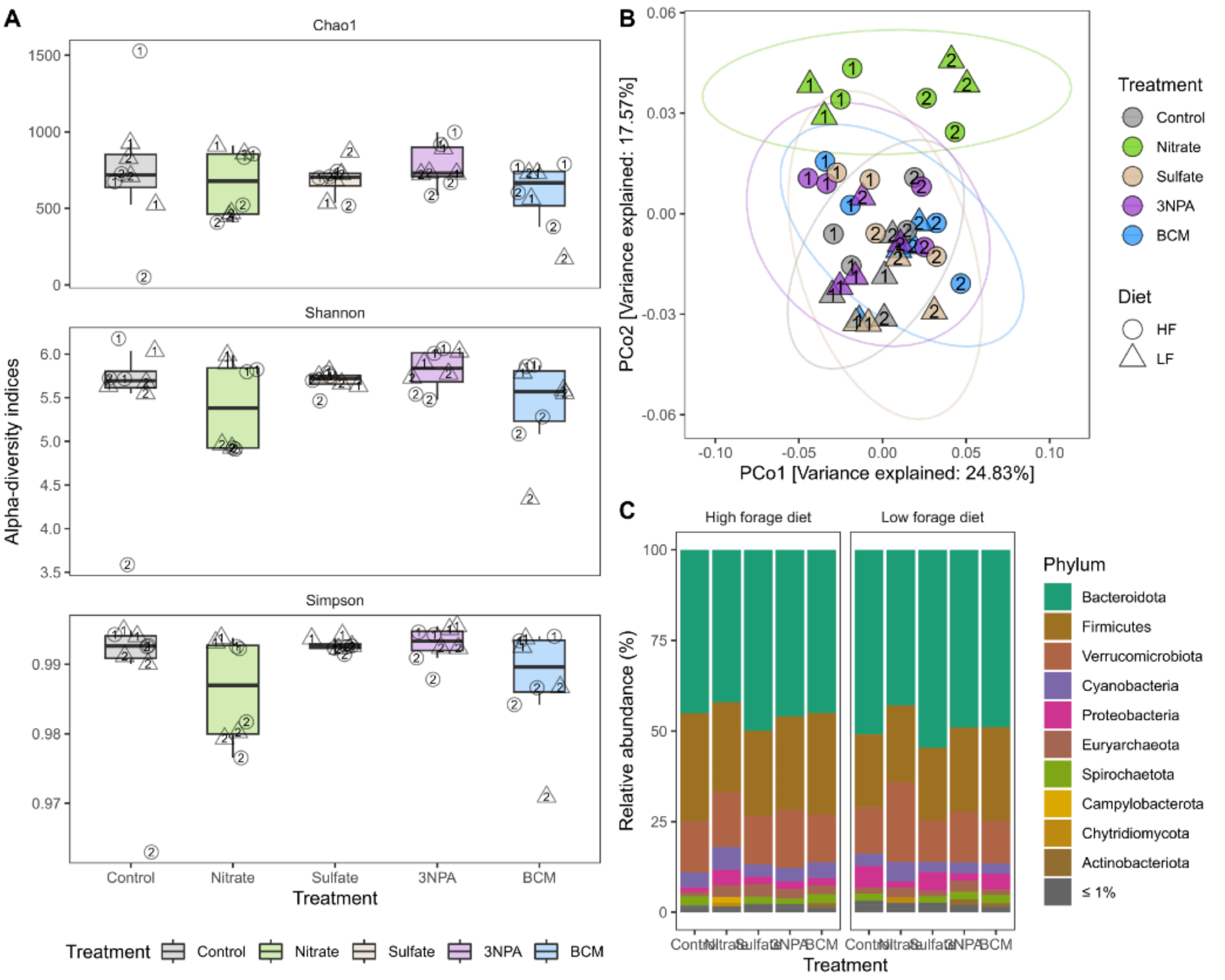

Supplementary Figure S1: Alpha- and beta-diversity measurements of samples from experiments 1 and 2. The treatments were as follows: Nitrate = 28 g/kg DM of sodium nitrate; Sulfate = 12 g/kg DM of sodium sulfate; 3NPA = 2.0 g/kg DM of 3-nitro-1-propionate; and BCM = 0.30 g/kg DM of bromochloromethane. The diets were high forage (HF) and low forage (LF) varying in forage to concentrate ratios of 70:30 and 30:70, respectively. (A) Chao1, Shannon, and Simpson diversity indices as measures of alpha-diversity or species richness and evenness; (B) principal component analysis of weighted UniFrac distance as a measure of beta-diversity or microbial community compositions; and (C) relative abundances of bacterial, archaeal, and fungal phyla. In Figures A and B, The number on each point correspond to the batch run 1 or 2.

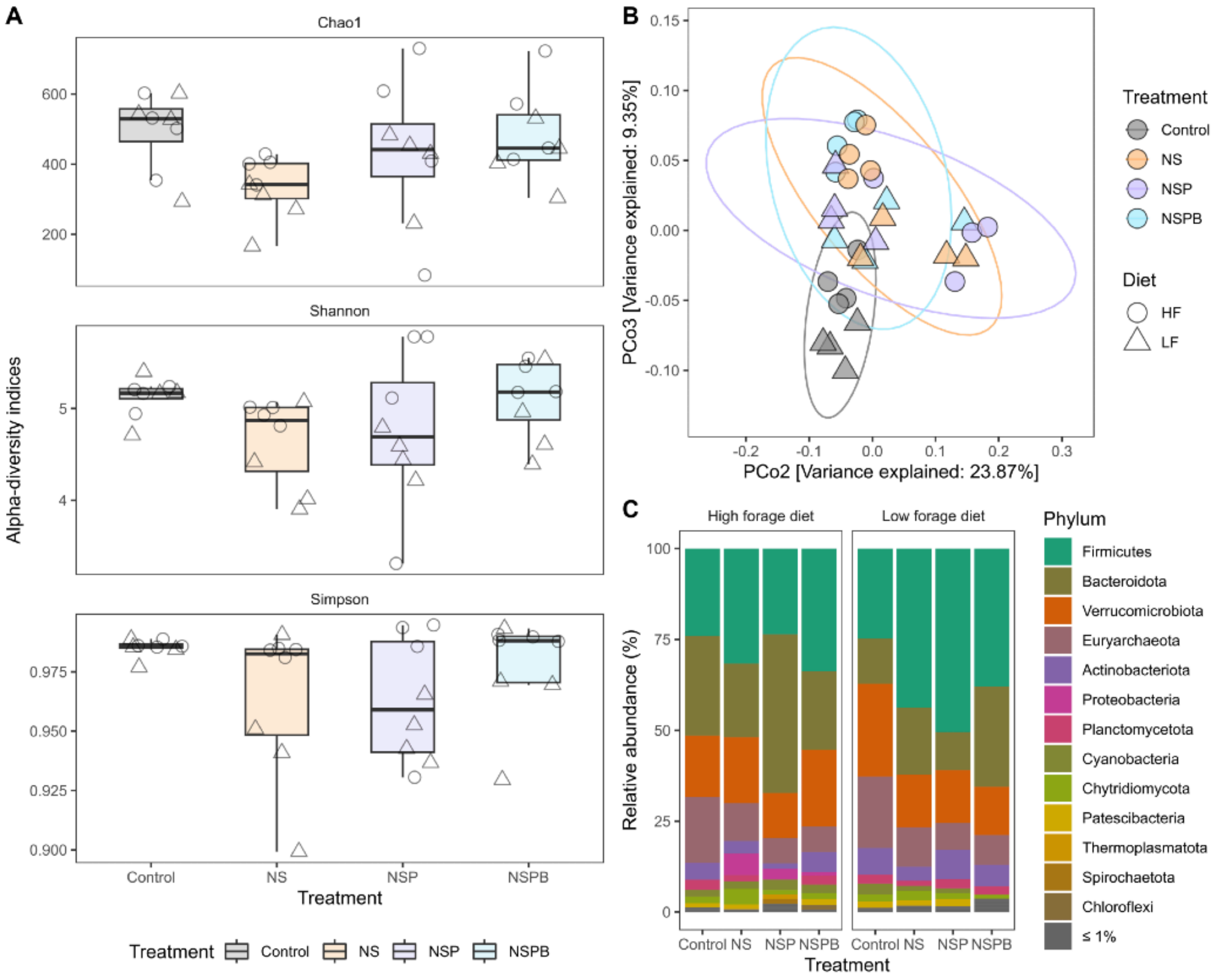

Supplementary Figure S2: Alpha- and beta-diversity measurements of samples from experiments 3 and 4. The treatments were as follows: NS = 28 g/kg DM of sodium nitrate + 12 g/kg DM of sodium sulfate; NSP = NS + 2.0 g/kg DM of 3-nitro-1-propionate; and NSPB = NSP + 0.30 g/kg DM of bromochloromethane. The diets were high forage (HF) and low forage (LF) varying in forage to concentrate ratios of 70:30 and 30:70, respectively. (A) Chao1, Shannon, and Simpson diversity indices as measures of alpha-diversity or species richness and evenness; (B) principal component analysis of Bray-Curtis dissimilarity as a measure of beta-diversity or microbial community compositions; (C) relative abundances of bacterial, archaeal, and fungal phyla.

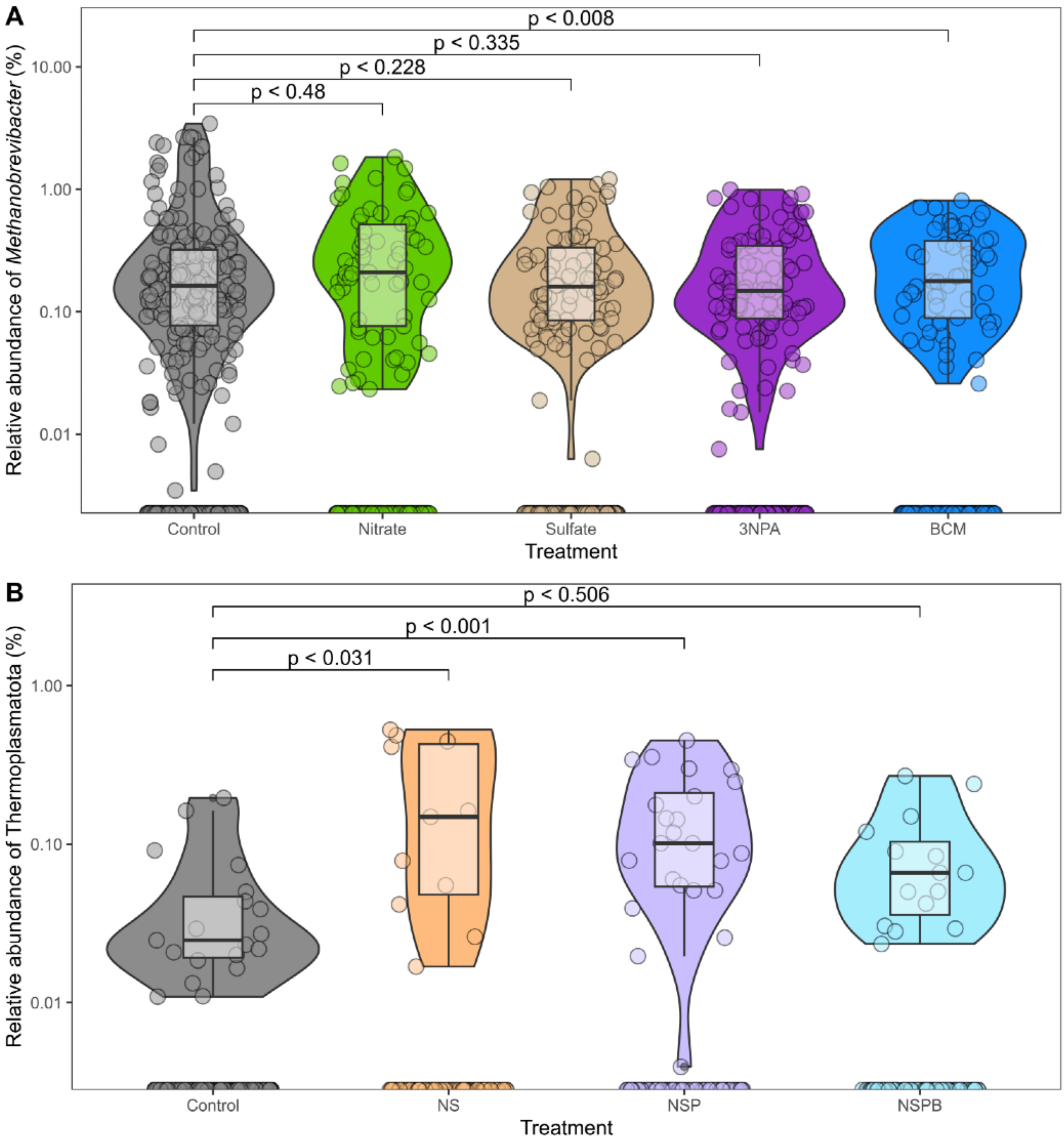

Supplementary Figure S3: Relative abundances of the genus *Methanobrevibacter* and the phylum Thermoplasmatota expressed as a percentage of the total amplicon sequences. (A) *Methanobrevibacter* relative abundance in the control cultures and those treated with sodium nitrate (Nitrate), sodium sulfate (Sulfate), 3-nitro-1-propionate (3NPA), and bromochloromethane (BCM) at 28, 12, 2.0, and 0.30 g/kg inclusion level on DM basis in experiments 1 and 2. (B) Thermoplasmatota relative abundance in the control and cultures treated with combinations of Nitrate + Sulfate (NS), NS + 3NPA (NSP), and NSP + BCM (NSPB) at the the above respective inclusion level in experiments 3 and 4.
